# Efficacy and safety of cannabinoids in the treatment of spasticity in multiple sclerosis: A systematic review of randomized clinical trials

**DOI:** 10.1002/bcp.70719

**Published:** 2026-08-02

**Authors:** Letícia Hamm‐Buiar, Daniela Gorski, Marcela Harumi Kagueiama, Astrid Wiens

**Affiliations:** ^1^ Federal University of Paraná (Universidade Federal do Paraná) Curitiba Brazil

**Keywords:** cannabinoids, multiple sclerosis, network meta‐analysis, spasticity, systematic review

## Abstract

**Aims:**

This work aimed to evaluate the efficacy and safety of cannabinoids for the treatment of MS‐related spasticity.

**Methods:**

Systematic searches were conducted in PubMed, EMBASE, and LILACS on 2 December 2025. Randomized controlled trials evaluating natural or synthetic cannabinoids for MS‐related spasticity were included. Studies published in non‐Roman characters and those assessing recreational cannabis use were excluded. Two reviewers independently performed study selection, data extraction, and risk of bias assessment using the RoB 2 tool. Qualitative synthesis, pairwise random‐effects meta‐analyses, and Bayesian network meta‐analysis were conducted. Certainty of evidence was assessed using GRADE and CINeMA.

**Results:**

Twenty‐seven studies (>3000 participants) were included. Interventions comprised tetrahydrocannabinol:cannabidiol (THC:CBD) extracts, other cannabis extracts, isolated cannabinoids (natural or synthetic), and smoked cannabis. In pairwise meta‐analysis (7 studies), THC:CBD extracts reduced patient‐reported spasticity versus placebo (MD −0.82; 95% CI −1.24 to −0.40; *I* ^2^ = 53%). Network meta‐analysis confirmed superiority of THC:CBD (MD −0.80; 95% CrI −1.61 to −0.32). Adverse events were frequent but mostly mild to moderate, while serious events were rare. Certainty of evidence was low, and substantial clinical and methodological heterogeneity was observed.

**Conclusions:**

Cannabinoids provide modest improvements in patient‐reported spasticity in MS, with more consistent effects for THC:CBD extracts. Given the low certainty of evidence and limited objective benefits, cannabinoids should be considered cautiously as adjunctive therapy with appropriate safety monitoring.

## 1 INTRODUCTION

Spasticity is a motor disorder characterized by a velocity‐dependent increase in tonic stretch reflexes (muscle tone), with exaggerated tendon spasms, resulting from hyperexcitability of the stretch reflex—a component of the upper motor neuron syndrome (Lance et al., 1980, cited in Trompetto et al.^1^). According to the consensus of the Interdisciplinary Movement Disorders Group, it can be defined as ‘involuntary muscle hyperactivity in the presence of central paresis’.^2^ Among neurological conditions associated with spasticity, multiple sclerosis (MS) stands out due to the high frequency of this symptom. It is estimated that 2.8 million people live with MS worldwide,^3^ and spasticity occurs in 60%–84% of patients over the course of the disease, being moderate to severe in 40%–60% of them.^4^ Spasticity contributes to impairment of mobility, dexterity, hygiene, self‐care, and the use of orthoses.^5^

In recent years, cannabinoids have attracted increasing interest as a therapeutic alternative for patients with MS. Published studies indicate that two natural components of the plant, tetrahydrocannabinol (THC) and cannabidiol (CBD), may be used for the treatment of spasticity associated with multiple sclerosis.^6^, ^7^, ^8^ The oromucosal spray containing the THC:CBD combination (nabiximols), for example, has been shown to be more effective than optimizing the dose of first‐line antispastic medications.^7^ Nabiximols, composed in a 1:1 ratio of THC and CBD, is already included in European guidelines for the management of spasticity in MS.^8^, ^9^, ^10^, ^11^ In international documents, its use is recommended as a second‐‐line,^9^, ^12^, ^13^ third‐‐line,^8^, ^10^, ^11^ or even fourth‐line pharmacological option,^14^ often before more invasive therapies such as intrathecal baclofen.^8^, ^11^, ^13^ Despite this progressive incorporation into clinical guidelines, there are still relevant uncertainties regarding the practical conduct of therapy, including selection criteria, response monitoring, and optimal treatment duration.^15^

In light of these uncertainties, this systematic review aimed to evaluate the efficacy and safety of cannabinoids in the treatment of spasticity in patients with multiple sclerosis, in order to critically synthesize the available evidence and provide support for clinical decision‐making and future therapeutic recommendations.

## 2 MATERIALS AND METHODS

This systematic review was conducted in accordance with the *Cochrane Handbook for Systematic Reviews of Interventions* ^16^ and reported following the *Preferred Reporting Items for Systematic Reviews and Meta‐Analyses* (PRISMA 2020).^17^ The PRISMA 2020 checklist is provided in Supporting Information. The protocol was registered in PROSPERO (CRD42024557313).

Randomized clinical trials evaluating the efficacy and safety of cannabinoids for the management of spasticity were included. Although the protocol had planned the inclusion of different clinical conditions and nonrandomized and observational studies, this manuscript reports randomized clinical trials conducted in patients with multiple sclerosis, due to the predominance of these studies in this population, allowing for a more homogeneous and concise synthesis.

The PICO strategy comprised the following:

P (participants)—patients with spasticity of any degree;

I (intervention)—natural or synthetic cannabinoids, without restriction regarding formulation, dose, or route of administration;

C (comparator)—placebo, antispastic therapies, usual care, or no exposure;

O (outcomes)—Primary outcomes: change in spasticity; change in complementary examinations assessing spasticity; range of motion; gait analysis; characterization of adverse events and serious adverse events (SAEs). Secondary outcomes: change in functional capacity; change in motor function; assessment of muscle strength; assessment of spasticity‐related pain; assessment of quality of life; withdrawals due to adverse events.

Studies published in non‐Roman characters, studies on recreational cannabis, and studies comparing different cannabis‐based treatments with each other were excluded. This last exclusion criterion had been previously protocolized and did not result in the exclusion of any studies, as no trials with these characteristics were identified. Thus, the body of included studies proved to be adequate for conducting a network meta‐analysis, as it presented a connected evidence network anchored in common comparators.

Searches were performed in PubMed, EMBASE, and LILACS on 2 December 2025. The search strategy included descriptors for spasticity and related diseases and cannabis‐based treatment, combined using the Boolean operators AND and OR (Supporting Information S1). In addition, a manual search was conducted in the reference lists of included studies and relevant systematic reviews identified.

### 2.1 Study selection

After duplicate removal, two independent reviewers screened titles and abstracts using the Rayyan platform. Subsequently, full texts of potentially eligible studies were assessed according to predefined criteria by the same two reviewers, independently. Disagreements were resolved by consensus or by a third reviewer.

### 2.2 Data extraction

Data extraction was performed independently by two reviewers using a standardized form (Microsoft Excel). Data were collected on study design, population, interventions, comparators, trial duration, methods, outcomes, and funding.

When the standard deviation of change was not reported, it was estimated from baseline and final values according to the *Cochrane Handbook*.^16^ The required correlation coefficient was empirically derived from the study by Fairhurst et al.^18^ Standard deviations obtained from 95% confidence intervals, standard errors, or *p* values were calculated using RevMan Web. Data presented only in graphical format were extracted using WebPlotDigitizer. The specific procedures adopted for each study are described in Supporting Information S2.

### 2.3 Risk of bias assessment

The risk of bias of randomized clinical trials was assessed by two reviewers using the Risk of Bias in Randomized Trials of Interventions tool (RoB 2.0).^19^

### 2.4 Data synthesis

#### 2.4.1 Qualitative synthesis

The characteristics of the populations, interventions, comparators, and main findings were described, highlighting the clinical, conceptual, and methodological heterogeneity.

#### 2.4.2 Pairwise meta‐analysis

When two or more studies were clinically comparable, a random‐effects meta‐analysis was conducted (meta package, R). Mean differences (MD) were calculated for continuous outcomes and risk ratios (RR) for dichotomous outcomes (95% CI). Heterogeneity was assessed using *I* ^2^ and explored through sensitivity analyses (leave‐one‐out).

#### 2.4.3 Network meta‐analysis

The included studies formed a connected evidence network, enabling a complementary NMA that was not prespecified in the PROSPERO protocol and was conducted without changes to the research question or eligibility criteria. The NMA was conducted in AutoNetBayes using arm‐level Bayesian models with noninformative priors. Convergence was assessed using trace plots, density plots, and the Brooks–Gelman–Rubin diagnostic. Results were presented as league tables (RR or MD with 95% CrI), with treatment rankings estimated by SUCRA and consistency evaluated through node‐splitting.

### 2.5 Certainty of the evidence

The certainty of the evidence was assessed using the GRADE system and its updates,^20^ considering the domains of risk of bias, inconsistency, indirectness, imprecision, and publication bias. For network comparisons, the certainty of network evidence was assessed using the CINeMA framework,^21^, ^22^ considering the domains of within‐study bias, reporting bias, indirectness, imprecision, heterogeneity, and incoherence. Despite differences in the domains assessed, both systems use the same scale to rate certainty of evidence: high, moderate, low, or very low.

### 2.6 Nomenclature of targets and ligands

Key protein targets and ligands in this article are hyperlinked to corresponding entries in http://www.guidetopharmacology.org and are permanently archived in the Concise Guide to PHARMACOLOGY 2025/26.^23^

## 3 RESULTS

The electronic search identified 3928 records in PubMed, EMBASE, and LILACS. After removal of 574 duplicates, 3354 records were submitted to title and abstract screening. Of these, 79 articles advanced to full‐text assessment.

Among the 79 studies assessed for eligibility, 52 were excluded for various reasons. All excluded articles, together with the respective reasons and full citations, are listed in Supporting Information S3.

At the eligibility stage, 25 studies met the pre‐established criteria and were included in the qualitative synthesis. In addition, two additional studies were identified through manual searching in other relevant systematic reviews, totalling 27 included studies.

The PRISMA flow diagram illustrating the process of study identification, selection, and inclusion is presented in Figure 1:

**FIGURE 1:**
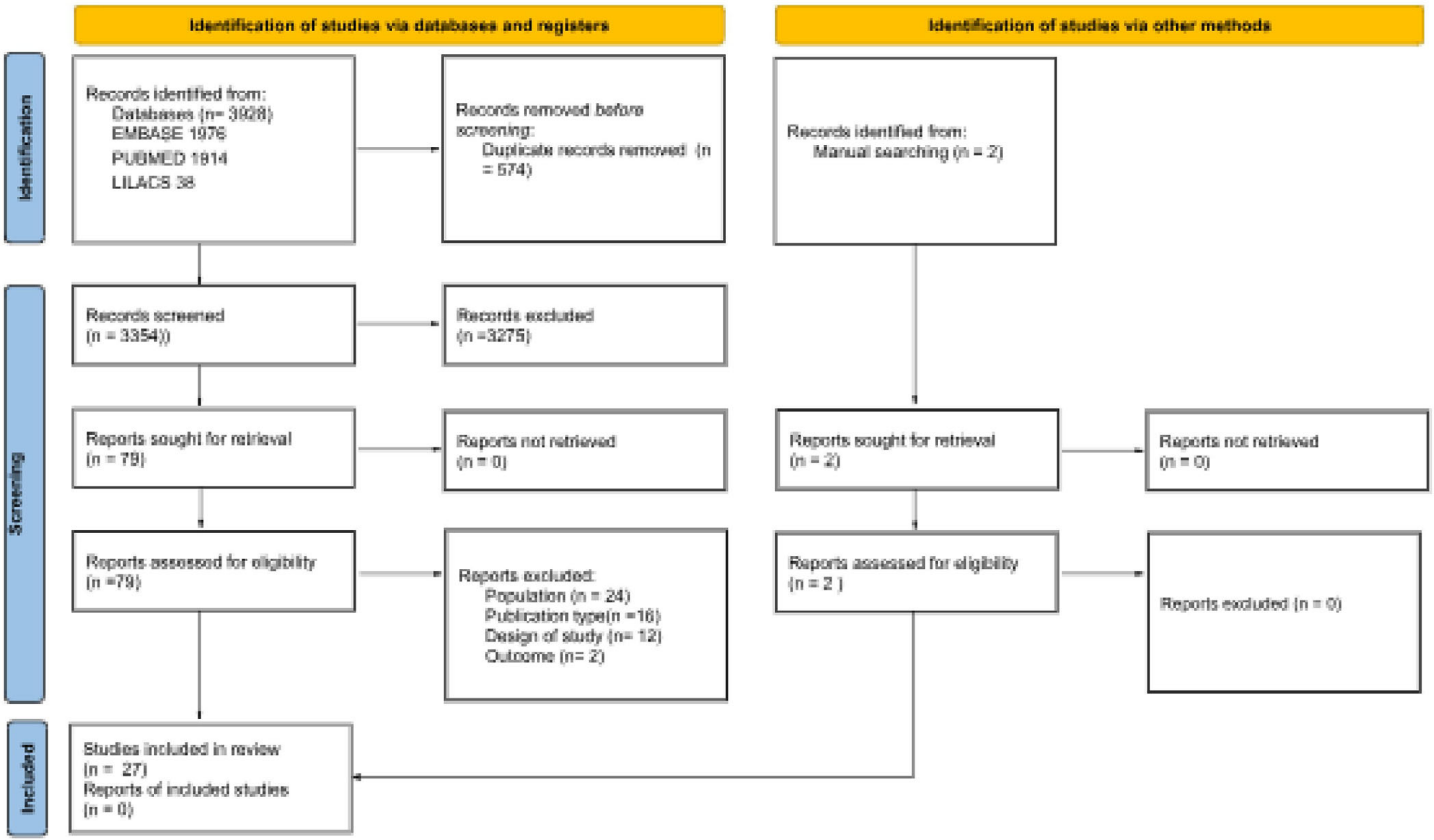
Flow diagram of selection of studies included in the systematic review, according to PRISMA 2020.

In total, 27 publications of randomized clinical trials were included, all involving patients with multiple sclerosis (MS). Two studies^24^, ^25^ also included participants with other neurological conditions; however, most of the samples comprised patients with MS (75% in study^24^ and 89% in study^25^). Thus, these studies were considered eligible for the present analysis.

The studies differed in design, evaluated formulations, and sample size. Older trials, generally small‐scale, investigated isolated THC or smoked cannabis, often using crossover designs. From the 2000s onward, greater methodological standardization was observed, with predominance of parallel, multicentre trials with larger samples, centred on the THC:CBD extract (nabiximols), which became the most studied formulation.

Study duration ranged from 2 weeks to 36 months. Almost all used placebo as the comparator, with the exception of Blasiis et al.,^26^ which used a nonexposed control group. The main methodological and clinical characteristics of the included studies are detailed in Table 1.

**TABLE 1:** Methodological and clinical characteristics of the included studies.

Author/year	Country	Study design	Clinical condition	Duration	Intervention	Comparator
Ungerleider et al., 1988^27^	United States	RCT, double‐blind, crossover	Multiple sclerosis	5 days per period	THC, *n* = 13	Placebo, *n* = 13
Killestein et al., 2002^28^	Netherlands	RCT, double‐blind, crossover, two phases	Multiple sclerosis	20 weeks	Dronabinol (THC), *n* = 16; cannabis extract, *n* = 16	Placebo, *n* = 16
Wade et al., 2003^24^	United Kingdom	RCT, double‐blind, crossover	Multiple sclerosis and other conditions^a^	4 weeks	THC extract, *n* = 20; CBD extract, *n* = 20; THC:CBD extract, *n* = 20	Placebo, *n* = 20
Zajicek et al., 2003^29^	United Kingdom	RCT, double‐blind, parallel, multicentre	Multiple sclerosis	15 weeks	Cannabis extract, *n* = 211; THC, *n* = 206	Placebo, *n* = 213
Vaney et al., 2004^30^	Switzerland	RCT, double‐blind, crossover	Multiple sclerosis	24 days	Cannabis extract, *n* = 57	Placebo, *n* = 57
Wade et al., 2004^31^	United Kingdom	RCT, double‐blind, parallel	Multiple sclerosis	6 weeks	THC:CBD extract, *n* = 80	Placebo, *n* = 80
Zajicek et al., 2005^32^	United Kingdom	RCT, double‐blind, multicentre	Multiple sclerosis	12 months	Cannabis extract, *n* = 211; THC, *n* = 206	Placebo, *n* = 213
Wissel et al., 2006^33^	Austria	RCT, double‐blind, crossover	Multiple sclerosis and other conditions^a^	9 weeks	Nabilone (THC), *n* = 13	Placebo, *n* = 13
Collin et al., 2007^34^	United Kingdom/Romania	RCT, double‐blind, multicentre	Multiple sclerosis	6 weeks	THC:CBD extract, *n* = 124	Placebo, *n* = 65
Aragona et al., 2009^35^	Italy	RCT, double‐blind	Multiple sclerosis	8 weeks	THC:CBD extract, *n* = 17	Placebo, *n* = 17
Conte et al., 2009^36^	Italy	RCT, double‐blind, crossover	Multiple sclerosis	15 weeks	THC:CBD extract, *n* = 18	Placebo, *n* = 18
Collin et al., 2010^37^	United Kingdom/Czech Republic	RCT, double‐blind, parallel	Multiple sclerosis	10 weeks	THC:CBD extract, *n* = 167	Placebo, *n* = 170
Notcutt et al., 2011^38^	United Kingdom	RCT, double‐blind, parallel, withdrawal	Multiple sclerosis	19 weeks	THC:CBD extract, *n* = 18	Placebo, *n* = 18
Novotna et al., 2011^39^	United Kingdom, Czech Republic, Spain, Poland, Italy	RCT, double‐blind, parallel, enriched	Multiple sclerosis	14 weeks	THC:CBD extract, *n* = 124	Placebo, *n* = 117
Corey‐Bloom et al., 2012^40^	Canada	RCT, double‐blind, crossover	Multiple sclerosis	2 weeks	Smoked cannabis cigarette, *n* = 37	Placebo, *n* = 37
Langford et al., 2012^41^	United Kingdom, Czech Republic, Canada, Spain, France	RCT, double‐blind, phase 3, parallel	Multiple sclerosis	14 weeks	THC:CBD extract, *n* = 167	Placebo, *n* = 172
Zajicek et al., 2012^42^	United Kingdom	RCT, double‐blind	Multiple sclerosis	10 weeks	Cannabis extract, *n* = 144	Placebo, *n* = 135
Tomassini et al., 2014^43^	Italy	RCT, double‐blind, crossover	Multiple sclerosis	10 weeks	THC:CBD extract, *n* = 18	Placebo, *n* = 18
Vachová et al., 2014^44^	Czech Republic	RCT, double‐blind, parallel	Multiple sclerosis	50 weeks	THC:CBD extract, *n* = 62	Placebo, *n* = 59
Ball et al., 2015^45^	England	RCT, double‐blind, parallel	Multiple sclerosis	36 months	Dronabinol (THC), *n* = 332	Placebo, *n* = 166
Leocani et al., 2015^46^	Italy	RCT, double‐blind, crossover	Multiple sclerosis	4 weeks	THC:CBD extract, *n* = 34	Placebo, *n* = 34
Van Amerongen et al., 2017^47^	Netherlands	RCT, double‐blind, parallel	Multiple sclerosis	13 weeks	Namisols (THC), *n* = 12	Placebo, *n* = 12
Markova et al., 2018^48^	Czech Republic/Austria	RCT, double‐blind, parallel	Multiple sclerosis	12 weeks	THC:CBD extract, *n* = 53	Placebo, *n* = 53
Blasiis et al., 2021^26^	Italy	RCT	Multiple sclerosis	4 weeks	THC:CBD extract, *n* = 22	Unexposed control, *n* = 10
Hansen et al., 2023^25^	Denmark	RCT, double‐blind, multicentre	MS and spinal cord injury^a^	7 weeks	Dronabinol (THC), *n* = 64; CBD, *n* = 62; dronabinol (THC) + CBD, *n* = 62	Placebo, *n* = 80
Bethoux et al., 2024^49^	Poland/Czech Republic	RCT, double‐blind, crossover	Multiple sclerosis	7 weeks	THC:CBD extract, *n* = 33	Placebo, *n* = 35
Mousavi et al., 2025^50^	Iran	RCT, double‐blind, parallel	Multiple sclerosis	4 weeks	CBD, *n* = 24	Placebo, *n* = 25

*Note*: RCT: randomized clinical trial; double‐blind: participants and investigators blinded; crossover: participants receive intervention and comparator in distinct periods; parallel: distinct groups; multicentre: more than one centre; phase 3: confirmatory trial; enriched: randomization restricted to responders; withdrawal: maintenance or withdrawal of treatment; n: number of participants per group. THC: tetrahydrocannabinol; CBD: cannabidiol; THC:CBD: combined formulation. MS: multiple sclerosis.

^a^ Studies including other neurological conditions in addition to MS.

### 3.1 Funding sources

Funding sources for the included trials were heterogeneous. Among the 27 included randomized clinical trial publications, 15 studies were sponsored wholly or partially by the pharmaceutical industry (including direct sponsorship, mixed funding, or provision of medication), especially those evaluating the standardized THC:CBD extract (nabiximols). Another eight studies received funding from public agencies, academic institutions, or philanthropic foundations, whereas in three studies funding was not clearly reported or could not be identified during the data extraction process, and one study explicitly declared that it did not receive specific funding. Funding sources are detailed in Supporting Information S4.

### 3.2 Efficacy assessment

#### 3.2.1 Primary outcome—Change in spasticity

Among the 27 included randomized clinical trial publications, most assessed spasticity using the Modified Ashworth Scale (MAS/Ashworth/cMAS/MAS‐LLMT) (*n* = 18), the Numerical Rating Scale (NRS) or Visual Analog Scale (VAS) (*n* = 16), or specific composite scales such as the Multiple Sclerosis Spasticity Scale (MSSS‐88) (*n* = 2). Individual trial results were initially synthesized descriptively, with presentation of effect direction by assessment instrument, in order to map response patterns between subjective and objective measures (Supporting Information S5). The meta‐analysis of the spasticity outcome assessed by the NRS scale included seven trials conducted in multiple sclerosis with THC:CBD extract compared with placebo and with continuous data or graphically extractable data.^24^, ^26^, ^34^, ^37^, ^39^, ^46^, ^48^ Some included studies could not be incorporated into meta‐analyses because they did not report complete numerical data and thus remained in the qualitative synthesis.

Figure 2 presents the endpoint meta‐analysis (NRS), in which an overall effect favourable to THC:CBD was observed.

**FIGURE 2:**
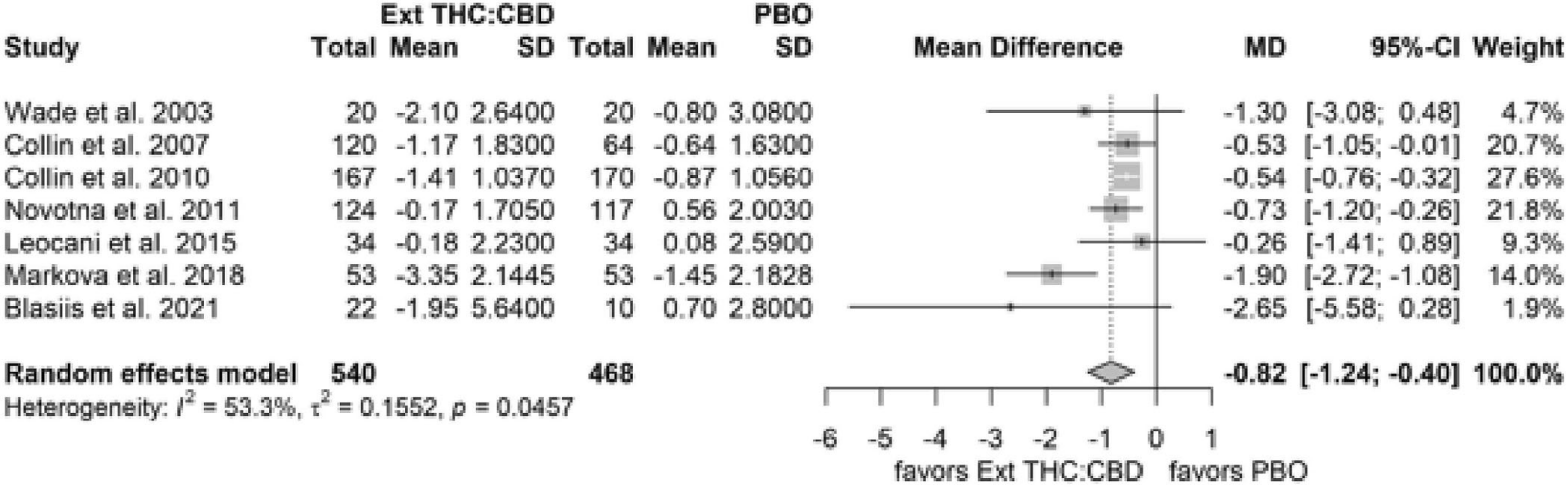
Pairwise random‐effects meta‐analysis comparing combined THC:CBD extract versus placebo (PBO) for self‐reported spasticity (NRS) in patients with multiple sclerosis. *Note*: Effects are expressed as mean difference (MD) with 95% confidence intervals (95% CI), calculated from subjective spasticity scales. Negative values favour treatment with THC:CBD extract. The diamond represents the pooled estimate, and individual squares reflect the statistical weight of each study. Heterogeneity among studies was moderate (*I* ^2^ = 53.3%; *p* = .0457).

The THC:CBD extract demonstrated a significant reduction in spasticity compared with placebo at the primary endpoint. Leave‐one‐out analyses showed that removal of any single study did not change the significance of the effect (Supporting Information S6), indicating robustness of the finding.

Heterogeneity was moderate to high (*I* ^2^ = 53.3%) and had as its main source the study by Markova et al.,^48^ which, when removed from the analysis, reduced heterogeneity to null. The trial by Markova et al. showed a substantially larger effect than the other studies, contributing meaningfully to heterogeneity. This study included only participants with prior response to THC:CBD and an individually optimized dose before randomization, resulting in greater contrast between the analysed arms.

In the 4‐week analyses, findings were consistent with the endpoint. In the pairwise random‐effects meta‐analysis, THC:CBD remained associated with a significant reduction in self‐reported spasticity compared with placebo, with moderate heterogeneity (*I* ^2^ = 50%; 5 studies), and leave‐one‐out analyses confirmed stability of the effect (Supporting Information S6).

Conducting additional direct meta‐analyses was not feasible due to heterogeneity among the evaluated formulations. Thus, a network meta‐analysis was performed, allowing indirect comparison among different treatments. Figure 3 presents the NMA comparing cannabis treatments assessed by the subjective NRS scale.

**FIGURE 3:**
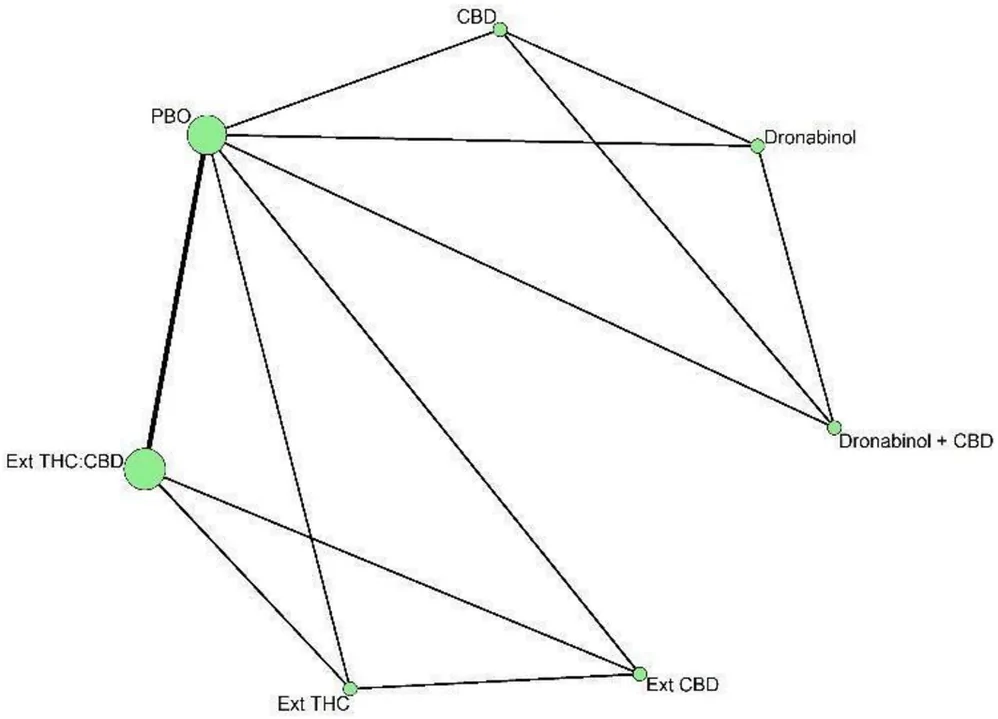
Network meta‐analysis of the effect of cannabinoids on self‐reported spasticity (NRS) in patients with multiple sclerosis. Abbreviations: PBO: placebo; CBD: cannabidiol; THC: tetrahydrocannabinol; Ext THC: THC‐rich extract; Ext CBD: CBD‐rich extract; Ext THC:CBD: standardized extract containing THC and CBD; dronabinol: synthetic THC. *Note*: Circle size is proportional to the total sample size of each intervention, while line thickness represents the number of studies contributing to each direct comparison.

Table 2 presents the league table of the network analysis for the spasticity endpoint. Only the THC:CBD extract demonstrated statistically significant superiority over placebo in the network analysis.

**TABLE 2:** Comparison table (league table) of the network meta‐analysis for self‐reported spasticity (NRS).

CBD						
0.12 (−1.68, 2.12)	Dronabinol					
0.25 (−1.73, 2.36)	0.07 (−1.98, 2.03)	Dronabinol___CBD				
1.67 (−1.05, 4.24)	1.56 (−1.19, 4.07)	1.42 (−1.34, 4.13)	Ext_CBD			
1.68 (−1.02, 4.44)	1.56 (−1.33, 4.37)	1.47 (−1.47, 4.32)	0.03 (−1.97, 2.09)	Ext_THC		
1.17 (−0.75, 3.21)	1.04 (−0.89, 3.00)	0.92 (−1.17, 3.17)	−0.47 (−2.41, 1.42)	−0.50 (−2.42, 1.56)	Ext_THC_CBD	
0.38 (−1.52, 2.12)	0.23 (−1.81, 1.92)	0.09 (−2.08, 2.29)	−1.30 (−3.28, 0.52)	−1.32 (−3.35, 0.62)	**−0.80 (−1.61, −0.32)**	PBO

*Note*: Results are presented as mean differences (MD) with 95% credibility intervals (95% CrI). Positive values favour the intervention listed in the row, and negative values favour the intervention listed in the column. Bold values indicate statistically significant comparisons (95% CrI not crossing 0). Interventions: CBD: cannabidiol; dronabinol: synthetic THC; dronabinol + CBD: combination of synthetic THC and CBD; Ext_CBD: CBD‐rich extract; Ext_THC: THC‐rich extract; Ext_THC_CBD: standardized extract containing THC and CBD; PBO: placebo.

The SUCRA analysis is presented in Supporting Information S7. Although isolated CBD and isolated THC occupied higher positions in the SUCRA ranking, this ordering reflects relative probabilities derived from the Bayesian model and not evidence of clinical superiority. The THC:CBD extract was the only treatment significantly superior to placebo in the NMA and in direct meta‐analysis.

### 3.3 Adverse events

Qualitative safety assessments across the 27 publications showed that cannabinoids were predominantly associated with mild to moderate adverse events (AEs). The most frequently reported AEs included dizziness, fatigue, somnolence, dry mouth, nausea, and a sensation of intoxication, especially in studies using isolated THC.

In trials evaluating isolated CBD or combined THC:CBD formulations (nabiximols), the overall incidence of adverse events was, in most studies, similar to that observed in placebo groups. Studies with nabiximols showed a consistent AE pattern, generally predictable and self‐limited, with a low rate of treatment‐related discontinuation. In studies using isolated THC, including dronabinol and Namisol, a higher reported frequency of neuropsychiatric adverse events was observed in qualitative synthesis, such as confusion, euphoria, mild cognitive changes, and transient worsening of balance. CBD‐rich formulations, evaluated in few more recent studies, showed a more favourable safety profile, with predominance of mild adverse events and rare psychoactive symptoms, although data availability remains limited. Inhaled cannabis, evaluated in a single trial, showed a distinct adverse event profile, characterized by immediate dizziness, mild euphoria, and transient respiratory discomfort.

SAEs were rare across the included trials and, in most cases, were not considered related to the intervention. The study with the largest number of participants (Ball et al., 2015)^45^ reported seven deaths (six in the active group and one in placebo), classified by investigators as resulting from disease progression or pre‐existing comorbidities, with no causal attribution to dronabinol use. Overall, no substantial differences in SAE occurrence were observed between active and placebo groups.

In more recent studies, particularly those conducted with isolated CBD, no SAEs or deaths were reported. In the trial by Mousavi et al.,^50^ discontinuations occurred due to mild events or lack of adherence to the protocol. Additional studies with nabiximols and full extracts also did not report SAEs attributable to treatment.

For the adverse events outcome, a network meta‐analysis was conducted. The NMA comparing the number of patients with adverse events associated with therapeutic cannabis use for the treatment of spasticity in multiple sclerosis and the corresponding league table are presented in Supporting Information S8. In the adverse events NMA, no formulation showed a statistically significant increase in adverse events compared with placebo.

Meta‐analyses of discontinuations and SAEs were not conducted due to the high proportion of studies with absence of events in one or both groups, especially in the placebo group. The low overall frequency of these outcomes, combined with heterogeneity among trials regarding formulation, treatment duration, and study population, could compromise stability and interpretation of pooled estimates.

#### 3.3.1 Secondary outcome—Spasticity‐related pain

Meta‐analysis of the spasticity‐related pain outcome was not performed due to wide heterogeneity of assessment instruments used across studies. Different scales and measurement methods were employed, including numerical and visual scales (NRS, VAS), self‐reports, composite measures, different analytical approaches (LOCF, BOCF, mixed models), and distinct assessment time points. This methodological variability would compromise comparability across studies and the validity of quantitative synthesis.

Collin et al.^37^ showed similar reductions between Sativex and placebo, indicating absence of a specific benefit. Conte et al.^36^ also found no significant effect of Sativex on post‐dose discomfort. In contrast, Corey‐Bloom et al.^40^ observed a clear improvement in pain with inhaled cannabis, one of the few trials with a consistent positive effect.

Hansen et al.^25^ reported very similar changes across THC, CBD, THC:CBD, and placebo, with no clinically relevant difference. Markovà et al.^48^ showed a greater reduction in the Sativex group than in placebo, suggesting possible benefit, although of limited magnitude. Van Amerongen et al.^47^ identified small improvements with nabiximols or oral Δ9‐THC, but also with modest differences versus placebo.

In older studies, Wade et al.^24^, ^31^ found small variations among different cannabis extracts and placebo, with no consistent direction. Wissel et al.^33^ was one of the few trials to demonstrate significant improvement with nabilone compared with placebo. Zajicek et al.^42^ reported only small differences between oral cannabis and placebo for the bodily pain outcome.

Overall, studies showed heterogeneous results, with some individual trials demonstrating pain reductions, while most showed modest effects or effects similar to placebo.

#### 3.3.2 Secondary outcome—Quality of life

Meta‐analysis of the quality‐of‐life outcome was not performed due to high heterogeneity of instruments used across studies, including generic and specific questionnaires (SF‐36, EQ‐5D, VASQoL), as well as diversity of domains assessed and forms of result presentation. In addition, many studies did not assess the outcome. Among trials reporting quality‐of‐life measures, results were predominantly small and without significant differences versus placebo. Aragona et al.^35^ showed a small reduction in VASQoL with both Sativex and placebo, with no difference between groups. Ball et al.^45^ observed no effect of dronabinol on the physical component of the SF‐36. Collin et al.^37^ found minimal changes in EQ‐5D and MSQoL‐54 domains, without statistical significance.

In Killestein et al. (2002),^28^ there was improvement in psychological domains of the SF‐36 during THC use but accompanied by worsening in the patient's global impression. Hansen et al.^25^ reported very small variations in EQ‐5D‐5L indices for THC, CBD, and THC:CBD, similar to placebo.

In older studies with cannabis extracts, Wade et al.^24^ observed small decreases in GHQ‐28 across different formulations (THC‐rich, CBD‐rich, and 1:1), with values close to placebo. Finally, Zajicek et al.^29^ found no evidence of effect on GHQ‐30.

#### 3.3.3 Secondary outcome—Functional capacity and motor function

It was not possible to conduct meta‐analysis for functional capacity and motor function outcomes due to extreme heterogeneity of instruments used across studies. Composite and MS‐specific scales were used (EDSS, MSFC, T25FW, 9‐Hole Peg Test, MSIS‐29, MSWS‐12, RMI), gait and balance tests (10‐m walk, 2MWT, 10MWT, TUG, BBS), activities of daily living indices (Barthel ADL), functional measures in other neurological conditions (ALSFRS‐R in ALS, Upper Motor Neuron Score), and cognitive‐functional tests (PASAT), often with different forms of analysis (time, score, relative or composite change).

Studies such as Aragona et al.,^35^ Ball et al.,^45^ Blasiis et al.,^26^ Collin et al.,^34^, ^37^ Corey‐Bloom et al.,^40^ Hansen et al.,^25^ Killestein et al.,^28^ Langford et al.,^41^ Markova et al.,^48^ Notcutt et al.,^38^ Novotna et al.,^39^ Van Amerongen et al.,^47^ Vaney et al.,^30^ Wade et al.,^24^, ^31^ Wissel et al.,^33^ and Zajicek et al.^29^, ^42^, ^45^ illustrate this diversity well, with emphasis on distinct combinations of outcomes and exploratory analyses or qualitative reports of no effect.

Overall, most studies did not demonstrate consistent benefit of cannabinoids on gait, mobility, manual dexterity, or performance in activities of daily living. When positive effects were described, they tended to be modest, restricted to specific domains or to secondary analyses. The coexistence of multiple scales, heterogeneous populations, and different forms of presentation prevents direct comparisons across trials and precludes a robust meta‐analysis for functional capacity.

Some individual trials reported small improvements in specific functional outcomes—such as balance (Blasiis et al.^26^), gait or physical performance (Corey‐Bloom et al.,^40^ Markova et al.,^48^), and mobility (Zajicek et al.^32^). However, these findings were isolated, not consistently reproduced, and, in general, of limited magnitude.

Despite being prespecified in the protocol, there was not sufficient and consistent data for analysis of range of motion, gait analysis, and muscle strength assessment.

### 3.4 Risk of bias assessment

Risk of bias assessment showed recurrent methodological limitations across included trials. Unclear risk was frequently observed in the randomization domain (D1) due to insufficient description of sequence generation and allocation concealment methods, as well as incomplete demographic information (D2), which hinder verification of baseline balance between groups. The outcome measurement domain (D3) was impacted by the use of subjective measures without fully blinding, increasing the possibility of detection bias. In addition, high or unbalanced attrition (D4–D5) was identified in several studies, often without appropriate analytical strategies; only a smaller proportion was judged as high risk, predominantly due to disproportionate losses, unavailable protocols, and inconsistencies between baseline and follow‐up measures. The complete risk of bias assessment is presented in Supporting Information S9.

### 3.5 Certainty of the evidence according to GRADE

Supporting Information S9 presents the certainty of evidence assessment using the GRADE method for the NRS outcome (THC:CBD *vs*. placebo). The evidence was downgraded in two domains—risk of bias and inconsistency—resulting in overall low certainty. Risk of bias was downgraded due to the predominance of studies with potential bias in subjective outcomes and important methodological limitations in one of the trials (crossover design, risk of carryover). Inconsistency was downgraded due to significant statistical heterogeneity and variability in effect estimates across studies. There was no downgrading for indirectness or imprecision.

### 3.6 Certainty of evidence according to CINeMA

The certainty of network evidence was assessed using the CINeMA (Confidence in Network Meta‐Analysis) framework. Among all comparisons analysed for the outcomes of spasticity assessed by the NRS and adverse events, expressed as the number of patients who experienced at least one adverse event, the comparison between combined THC:CBD extract and placebo showed low certainty of evidence regarding the effect estimate, mainly due to the substantial heterogeneity observed. The other comparisons were also rated as low certainty of evidence, chiefly due to major concerns related to imprecision, stemming from the limited number of studies per comparison and the wide credibility intervals. In some comparisons, concerns regarding indirectness were also identified, associated with differences in populations, routes of administration, and study designs included in the network. The complete certainty of evidence assessment is presented in Supporting Information S9.

## 4 DISCUSSION

The findings of this systematic review indicate that cannabinoids, particularly the standardized THC:CBD extract, exert a modest yet consistent effect in reducing self‐reported spasticity in patients with multiple sclerosis, as demonstrated in the endpoint meta‐analysis and in analyses conducted after 4 weeks of treatment. Similarly to what has been reported in previous systematic reviews, the benefit is more evident in subjective measures (e.g., NRS), whereas objective scales of muscle tone, such as the Modified Ashworth Scale, showed limited responsiveness and frequently nonsignificant results.^51^, ^52^, ^53^ This pattern suggests a predominant effect on symptom perception rather than on measurable muscle tone.

However, the discrepancy between patient‐reported improvement in spasticity and objective measures should also be interpreted considering the methodological limitations of currently available spasticity assessment tools in multiple sclerosis. The Modified Ashworth Scale, although widely used in clinical practice and research, has recognized psychometric limitations, including its ordinal nature, limited sensitivity to change, variability in interrater reliability, and difficulty distinguishing neural components of spasticity from nonneural biomechanical factors such as stiffness and contracture. Similar methodological challenges have also been described for other commonly used clinical scales, particularly considering the multidimensional and fluctuating characteristics of spasticity in multiple sclerosis. In this context, multidimensional instruments such as the Multiple Sclerosis Spasticity Scale‐88 may provide a broader assessment of patient experience and functional impact.^4^

Despite these methodological limitations, the overall pattern of findings remains consistent with current clinical recommendations. The consistency of these findings is also reflected in international guidelines, particularly the NICE NG144 guideline,^6^ which recommends the use of THC:CBD only as adjuvant therapy for moderate to severe spasticity, with discontinuation of treatment if no clinically relevant response is observed after a short trial period. The present review is consistent with this guidance by integrating the designs of some trials with responder‐enrichment phases and the heterogeneity observed in effect estimates, suggesting that part of the benefit may be concentrated in patients who show an initial clinical response.

In contrast to previous reviews that included observational studies, open‐label extensions, or noncomparator phases and prioritized dichotomous response outcomes,^54^ the present review was restricted to comparative randomized clinical trials and used continuous measures of self‐reported spasticity (NRS). This approach allowed estimation of the causal effect relative to placebo and reduced the risk of overestimation associated with selected populations, resulting in a more conservative and clinically delimited interpretation of cannabinoid efficacy.

A recent systematic review concluded that nabiximols is the most widely used and most effective cannabinoid for controlling spasticity in patients with multiple sclerosis; however, most included studies were based on noncomparative designs, which limits the robustness of those conclusions.^55^ In this context, the present review contributes by restricting inclusion to comparative randomized clinical trials conducted exclusively in multiple sclerosis, thereby reducing conceptual heterogeneity and increasing methodological consistency.

Despite the substantial number of studies included in this review, methodological limitations persist, such as insufficient description of randomization methods and allocation concealment, the predominant use of subjective outcomes in contexts in which blinding may have been partially compromised, relevant attrition, and crossover designs with risk of carryover. Heterogeneity among instruments used to assess spasticity, dose titration schemes, follow‐up duration, and participant selection criteria contributes to the observed variability and explains the downgrading of certainty of evidence in the GRADE assessment.

The difference observed between certainty of evidence classifications by GRADE and CINeMA reflects the distinct dimensions evaluated by these frameworks. While GRADE emphasizes methodological limitations of primary studies and clinical consistency of results, CINeMA focuses on confidence in estimates derived from network meta‐analysis, considering network structure, the contribution of direct and indirect evidence, and estimate precision. Thus, the low certainty of evidence classification by both CINeMA and GRADE suggests that, although the network meta‐analysis shows internal statistical consistency, clinical interpretation of the effects should be cautious, given the quality and methodological characteristics of the included studies.

Regarding safety, the findings of this study indicate that the adverse event profile associated with therapeutic cannabinoid use in patients with multiple sclerosis is generally consistent with the pharmacological profile of these substances, with variations depending on formulation, dose, and, to a lesser extent, treatment duration. This pattern is in line with previous systematic reviews and evidence‐based guidelines, which describe an overall increase in treatment‐related adverse events, usually without clinically relevant severity.^6^, ^52^, ^56^

Fixed‐ratio THC:CBD formulations, particularly standardized extracts used in nabiximols trials, have been associated with better tolerability, with lower occurrence of psychoactive symptoms and relatively low discontinuation rates. A possible mechanism underlying this more favourable profile is the modulatory effect of CBD on the psychoactive effects of THC, a hypothesis supported by systematic syntheses and by the evidence review underpinning the NICE guideline, which indicates greater tolerability of the THC:CBD oromucosal spray compared with formulations containing isolated THC.^6^, ^52^

Conversely, accumulated evidence in the literature suggests that formulations containing isolated THC, such as dronabinol or predominantly tetrahydrocannabinolic extracts, present a less favourable tolerability profile, with greater impact on the central nervous system and higher risk of discontinuation. Previous reviews reinforce this association, indicating that isolated THC exposure is an important determinant of adverse event occurrence and treatment adherence.^52^, ^53^, ^56^

CBD‐rich formulations, evaluated in a limited number of more recent studies, suggest a potentially more favourable safety profile, although data scarcity still precludes definitive conclusions. Similarly, smoked cannabis, evaluated in a single trial,^40^ presents a distinct adverse event profile, the interpretation of which should consider not only cannabinoid effects but also the inhalation route of administration, as highlighted in previous syntheses.

SAEs remained rare across the different formulations evaluated and, when observed, tended to occur in populations with more severe disease, which complicates direct causal attribution to treatment. This pattern is consistent with previous systematic reviews and reinforces that clinical use of cannabinoids in multiple sclerosis involves a risk–benefit balance strongly influenced by the formulation used and the dose titration strategy.^6^, ^23^, ^52^

Overall, studies suggest that cannabinoids may alleviate spasticity‐related pain in some specific scenarios; however, in general, observed effects were modest and often similar to placebo. Positive findings were concentrated in a few individual trials, particularly those evaluating inhaled cannabis or nabilone, whereas most studies with oral extracts or nabiximols showed discrete or inconsistent results, in agreement with previous systematic reviews.^52^, ^53^, ^56^

This heterogeneity may reflect relevant differences among studies in formulation, route of administration, included populations, and instruments used to assess pain. In addition, conceptual overlap between pain, spasticity, and motor discomfort in patients with multiple sclerosis may hinder identification of a specific analgesic effect, an aspect also highlighted in previous syntheses.^52^, ^56^

Overall, results indicate that cannabinoid treatment does not consistently translate into improvement in global quality of life, even in trials in which reductions in specific symptoms such as spasticity or pain were observed. Improvements were small, often restricted to isolated domains and, in some cases, accompanied by worsening in other subjective measures, such as patient global impression—findings largely consistent with systematic reviews and assessments used in clinical guidelines.^6^, ^52^

These results suggest that focal symptomatic benefits do not always translate into perceived functional or psychosocial gains, possibly due to short study duration, coexistence of mild adverse events, or the natural progression of the disease. The discrepancy between specific scales and global quality‐of‐life measures further limits clinical interpretation of these findings, as previously discussed in the literature.^53^, ^56^

Interpretation of cannabinoid effects on functional capacity and motor function is hampered by marked methodological heterogeneity among included trials. The coexistence of multiple scales, distinct clinical populations, and different forms of result presentation prevents direct comparisons and limits quantitative synthesis, a pattern also described in previous systematic reviews.^52^, ^56^

Although some individual studies reported isolated improvements in specific functional outcomes, these effects were small in magnitude and not consistently reproduced. Thus, both the present review and prior literature converge on the absence of robust evidence of clinically relevant functional benefit associated with cannabinoid use in patients with spasticity related to multiple sclerosis.^52^, ^53^

### 4.1 Contributions and implications

This review incorporates recent evidence (up to 2025) not included in reviews used by current guidelines, performing a direct meta‐analysis of spasticity measured by NRS restricted to multiple sclerosis and a network meta‐analysis comparing multiple cannabinoid formulations. This approach allowed identification that only the standardized THC:CBD extract shows consistent benefit compared with placebo, whereas other formulations exhibit uncertain or weak efficacy.

From a clinical perspective, these findings suggest that patient‐perceived spasticity is more responsive to cannabinoids than muscle tone measured by clinical scales. However, this difference may also reflect important limitations of the instruments commonly used to assess spasticity in multiple sclerosis. Therefore, the inconsistency between patient‐reported improvement and objective measures may partly result from limitations of current assessment tools.

However, the absence of consistent improvement in motor and functional scales indicates that, although cannabinoids may be useful as adjuvant therapy for specific symptoms, there is no robust evidence of clinically relevant functional benefit in the available studies.

### 4.2 Limitations and suggestions for future research

Included trials present important methodological limitations that restrict the strength of conclusions. Heterogeneity across formulations, doses, durations, and assessment instruments complicates comparative synthesis and precludes robust meta‐analyses for several outcomes. In addition, many studies have small sample sizes, relevant attrition during follow‐up, potential compromise of blinding for subjective outcomes, and short follow‐up duration.

One limitation of this review is that the network meta‐analysis was not pre‐specified in the registered PROSPERO protocol. Nevertheless, it was conducted as a complementary analysis based on the same eligibility criteria and research question defined a priori.

An additional limitation is the geographic concentration of available evidence. Most randomized clinical trials were conducted in European countries, with limited representation from other regions, which may restrict generalizability of findings to different healthcare systems, regulatory contexts, and patient populations.

Future research should prioritize methodologically robust randomized clinical trials with clear description of randomization and allocation concealment processes and adequate blinding. Longer‐duration studies are needed to assess maintenance of effect and long‐term safety, as well as investigations exploring different THC and CBD proportions and identifying clinical profiles associated with greater likelihood of response, contributing to a more individualized treatment approach.

## 5 CONCLUSION

This systematic review demonstrates, through meta‐analysis, that cannabinoids—particularly the standardized THC:CBD oromucosal spray—exert a modest yet consistent effect in reducing self‐reported spasticity in patients with multiple sclerosis. In contrast, the impact on objective measures of muscle tone and functional outcomes was more limited, as evidenced by the qualitative synthesis of the included studies. In addition, cannabinoid use was associated with mild to moderate adverse events, while SAEs were rare and generally not clearly attributable to treatment.

Considering the overall low certainty of the evidence and the methodological limitations of the available trials, cannabinoids should be used with caution in the management of spasticity associated with multiple sclerosis and may be considered as an adjuvant option aimed at improving patient‐perceived symptoms, within the context of shared decision‐making and safety monitoring.

## AUTHOR CONTRIBUTIONS

Letícia Hamm‐Buiar conceived and designed the study, developed the protocol, conducted the literature search, served as the first reviewer at all stages of the review, interpreted the results, and drafted the manuscript. Marcela Harumi Kagueiama served as the second reviewer during study selection and data extraction. Daniela Gorski planned and performed the statistical analyses and served as the second reviewer for the risk of bias assessment. Astrid Wiens contributed to methodological guidance, study supervision, and critical revision of the manuscript.

## CONFLICT OF INTEREST STATEMENT

The authors declare no conflicts of interest.

## Supporting information


**Data S1.** Search terms used.
Data S2. Data extraction and imputation procedures for the meta‐analysis.
Data S3. Excluded studies in elegibility.
Data S4. Funding sources of the included studies.
**Data S5.** Individual results of randomized clinical trials evaluating cannabinoids for the treatment of spasticity in patients with multiple sclerosis, presented according to the assessment instrument used.
Data S6. Meta‐analysis plots and sensitivity analyses (leave‐one‐out).
Data S7. Network meta‐analysis.
Data S8. Risk of bias assessment of the included trials by outcome, according to the Risk of Bias 2.0 (RoB 2.0) tool.
Data S9. Certainty of evidence.

## Data Availability

Data extraction forms, spreadsheets containing data extracted from the included studies, and detailed analytical results are available from the corresponding authors upon reasonable request.
