# Supplementary material for: Efficacy and safety of cannabinoids in the treatment of spasticity in multiple sclerosis: A systematic review of randomized clinical trials
Source: Br J Clin Pharmacol. 2026 Aug 2;92(10):3288–300. doi: 10.1002/bcp.70719 (PMC13619024; doi:10.1002/bcp.70719)
Supplement: Supplementary file 1 [file BCP-92-3288-s001.docx]

**PRISMA 2020 Main Checklist**

| **Topic** | **No.** | **Item** | **Location where item is reported** |
| --- | --- | --- | --- |
| **TITLE** |  |  |  |
| **Title** | 1 | Identify the report as a systematic review. | p. 1 |
| **ABSTRACT** |  |  |  |
| **Abstract** | 2 | See the PRISMA 2020 for Abstracts checklist |  |
| **INTRODUCTION** |  |  |  |
| **Rationale** | 3 | Describe the rationale for the review in the context of existing knowledge. | pp. 3-4 |
| **Objectives** | 4 | Provide an explicit statement of the objective(s) or question(s) the review addresses. | p. 4 |
| **METHODS** |  |  |  |
| **Eligibility criteria** | 5 | Specify the inclusion and exclusion criteria for the review and how studies were grouped for the syntheses. | p. 5 |
| **Information sources** | 6 | Specify all databases, registers, websites, organisations, reference lists and other sources searched or consulted to identify studies. Specify the date when each source was last searched or consulted. | p. 6 |
| **Search strategy** | 7 | Present the full search strategies for all databases, registers and websites, including any filters and limits used. | Supplementary Material 1 |
| **Selection process** | 8 | Specify the methods used to decide whether a study met the inclusion criteria of the review, including how many reviewers screened each record and each report retrieved, whether they worked independently, and if applicable, details of automation tools used in the process. | p. 6 |
| **Data collection process** | 9 | Specify the methods used to collect data from reports, including how many reviewers collected data from each report, whether they worked independently, any processes for obtaining or confirming data from study investigators, and if applicable, details of automation tools used in the process. | p. 6 |
| **Data items** | 10a | List and define all outcomes for which data were sought. Specify whether all results that were compatible with each outcome domain in each study were sought (e.g. for all measures, time points, analyses), and if not, the methods used to decide which results to collect. | p. 5 |
|  | 10b | List and define all other variables for which data were sought (e.g. participant and intervention characteristics, funding sources). Describe any assumptions made about any missing or unclear information. | p. 7 |
| **Study risk of bias assessment** | 11 | Specify the methods used to assess risk of bias in the included studies, including details of the tool(s) used, how many reviewers assessed each study and whether they worked independently, and if applicable, details of automation tools used in the process. | p. 7 |
| **Effect measures** | 12 | Specify for each outcome the effect measure(s) (e.g. risk ratio, mean difference) used in the synthesis or presentation of results. | p. 7 |
| **Synthesis methods** | 13a | Describe the processes used to decide which studies were eligible for each synthesis (e.g. tabulating the study intervention characteristics and comparing against the planned groups for each synthesis (item 5)). | pp. 5 and 7 |
|  | 13b | Describe any methods required to prepare the data for presentation or synthesis, such as handling of missing summary statistics, or data conversions. | p. 6 and Supplementary Material 2 |
|  | 13c | Describe any methods used to tabulate or visually display results of individual studies and syntheses. | pp. 5 and 7 |
|  | 13d | Describe any methods used to synthesize results and provide a rationale for the choice(s). If meta-analysis was performed, describe the model(s), method(s) to identify the presence and extent of statistical heterogeneity, and software package(s) used. | p. 7 |
|  | 13e | Describe any methods used to explore possible causes of heterogeneity among study results (e.g. subgroup analysis, meta-regression). | p. 7 |
|  | 13f | Describe any sensitivity analyses conducted to assess robustness of the synthesized results. | p. 7 |
| **Reporting bias assessment** | 14 | Describe any methods used to assess risk of bias due to missing results in a synthesis (arising from reporting biases). | p. 7 |
| **Certainty assessment** | 15 | Describe any methods used to assess certainty (or confidence) in the body of evidence for an outcome. | p. 7 |
| **RESULTS** |  |  |  |
| **Study selection** | 16a | Describe the results of the search and selection process, from the number of records identified in the search to the number of studies included in the review, ideally using a flow diagram. | pp. 8-9 |
|  | 16b | Cite studies that might appear to meet the inclusion criteria, but which were excluded, and explain why they were excluded. | Supplementary material 3 |
| **Study characteristics** | 17 | Cite each included study and present its characteristics. | pp. 8-12 |
| **Risk of bias in studies** | 18 | Present assessments of risk of bias for each included study. | Supplementary Material 8 |
| **Results of individual studies** | 19 | For all outcomes, present, for each study: (a) summary statistics for each group (where appropriate) and (b) an effect estimate and its precision (e.g. confidence/credible interval), ideally using structured tables or plots. | pp. 13-21 |
| **Results of syntheses** | 20a | For each synthesis, briefly summarise the characteristics and risk of bias among contributing studies. | pp. 13-21 |
|  | 20b | Present results of all statistical syntheses conducted. If meta-analysis was done, present for each the summary estimate and its precision (e.g. confidence/credible interval) and measures of statistical heterogeneity. If comparing groups, describe the direction of the effect. | pp. 13-21 and Supplementary Materials 6 and 7 |
|  | 20c | Present results of all investigations of possible causes of heterogeneity among study results. | pp.13-14 and Supplementary Material 6 |
|  | 20d | Present results of all sensitivity analyses conducted to assess the robustness of the synthesized results. | Supplementary Material 6 |
| **Reporting biases** | 21 | Present assessments of risk of bias due to missing results (arising from reporting biases) for each synthesis assessed. | p. 20 and Supplementary Material 8 |
| **Certainty of evidence** | 22 | Present assessments of certainty (or confidence) in the body of evidence for each outcome assessed. | p. 21 and Supplementary material 9 |
| **DISCUSSION** |  |  |  |
| **Discussion** | 23a | Provide a general interpretation of the results in the context of other evidence. | pp. 21-25 |
|  | 23b | Discuss any limitations of the evidence included in the review. | p. 25 |
|  | 23c | Discuss any limitations of the review processes used. | p. 25 |
|  | 23d | Discuss implications of the results for practice, policy, and future research. | pp. 25-26 |
| **OTHER INFORMATION** |  |  |  |
| **Registration and protocol** | 24a | Provide registration information for the review, including register name and registration number, or state that the review was not registered. | p. 2 |
|  | 24b | Indicate where the review protocol can be accessed, or state that a protocol was not prepared. | p. 2 |
|  | 24c | Describe and explain any amendments to information provided at registration or in the protocol. | pp. 4 and 6 |
| **Support** | 25 | Describe sources of financial or non-financial support for the review, and the role of the funders or sponsors in the review. | p. 27 |
| **Competing interests** | 26 | Declare any competing interests of review authors. | p. 27 |
| **Availability of data, code and other materials** | 27 | Report which of the following are publicly available and where they can be found: template data collection forms; data extracted from included studies; data used for all analyses; analytic code; any other materials used in the review. | p. 27 |

*From:* Page MJ, McKenzie JE, Bossuyt PM, Boutron I, Hoffmann TC, Mulrow CD, et al. The PRISMA 2020 statement: an updated guideline for reporting systematic reviews. MetaArXiv. 2020, September 14. DOI: 10.31222/osf.io/v7gm2. For more information, visit: [www.prisma-statement.org](about:blank)

**Supplementary Material 1 – Search terms used**

**MEDLINE (Pubmed) #1 AND #2**

#1 ("Cannabis"[Mesh] OR "Cannabidiol"[Mesh] OR "Cannabinoids"[Mesh] OR "Medical Marijuana"[Mesh] OR "Medical Marijuana”[TIAB] OR "Cannabis"[TIAB] OR “Cannabidiol”[TIAB] OR "Cannabinoids"[TIAB] OR "Marijuana, Medical"[TIAB] OR "Medicinal Marijuana"[TIAB] OR "Marijuana, Medicinal"[TIAB] OR "Cannabi"[TIAB] OR "Tetrahydrocannabinol"[TIAB] OR "Cannabinol"[TIAB] OR "Cannabidiolic"[TIAB] OR "Epidiolex"[TIAB] OR “Nabiximols”[TIAB] OR “Nabilone”[TIAB] OR “Dronabinol*”[TIAB] OR “Cannabigerol*”[TIAB] OR “Cannabidivarin”[TIAB] OR “Cannabichromene*”[TIAB] OR “Bedrobinol”[TIAB] OR “THC”[TIAB] OR “Tetrahydrocannabivarin*”[TIAB] OR “CBD”[TIAB] OR “Tetrahydrocannabinol*”[TIAB])

#2 ("Muscle Spasticity"[Mesh] OR "Muscle Hypertonia"[Mesh] OR "Muscle Spasticity"[TIAB] OR "Muscle Hypertonia” [TIAB] OR “Hypertonia”[TIAB] OR “Muscle Rigity”[TIAB] OR “Spasticity, Muscle”[TIAB] OR “Spastic Clasp-Knife Spasticity”[TIAB] OR “Clasp Knife Spasticity”[TIAB] OR “Spasticity, Clasp-Knife”[TIAB] OR “spastic* or high tone”[TIAB] OR "Multiple Sclerosis"[Mesh] OR "Multiple Sclerosis"[TIAB] OR “Sclerosis, Multiple”[TIAB] OR “Sclerosis, Disseminated”[TIAB] OR “Disseminated Sclerosis”[TIAB] OR “Multiple Sclerosis, Acute Fulminating”[TIAB] OR "Cerebral Palsy"[Mesh] OR “Cerebral Palsy”[TIAB] OR “Spastic Diplegia”[TIAB] OR “Diplegias, Spastic”[TIAB] OR “Spastic Diplegias”[TIAB] OR “Diplegia, Spastic”[TIAB] OR “Cerebral Palsy, Spastic”[TIAB] OR “Spastic Cerebral Palsies”[TIAB] OR “Spastic Cerebral Palsy”[TIAB] OR “Cerebral Palsy, Dystonic-Rigid”[TIAB] OR “Cerebral Palsies, Dystonic-Rigid”[TIAB] OR “Cerebral Palsy, Dystonic Rigid”[TIAB] OR “Dystonic-Rigid Cerebral Palsies”[TIAB] OR “Dystonic-Rigid Cerebral Palsy”[TIAB] OR “Cerebral Palsy, Mixed”[TIAB] OR “Mixed Cerebral Palsies”[TIAB] OR “Mixed Cerebral Palsy”[TIAB] OR “Cerebral Palsy, Monoplegic, Infantile”[TIAB] OR “Monoplegic Infantile Cerebral Palsy”[TIAB] OR “Infantile Cerebral Palsy, Monoplegic”[TIAB] OR “Cerebral Palsy, Quadriplegic, Infantile”[TIAB] OR “Quadriplegic Infantile Cerebral Palsy”[TIAB] OR “Infantile Cerebral Palsy, Quadriplegic”[TIAB] OR “Cerebral Palsy, Rolandic Type”[TIAB] OR “Rolandic Type Cerebral Palsy”[TIAB] OR “Cerebral Palsy, Congenital”[TIAB] OR “Congenital Cerebral Palsy”[TIAB] OR “Little Disease”[TIAB] OR “Little's Disease”[TIAB] OR “Monoplegic Cerebral Palsy”[TIAB] OR “Cerebral Palsies, Monoplegic”[TIAB] OR “Cerebral Palsy, Monoplegic”[TIAB] OR “Monoplegic Cerebral Palsies”[TIAB] OR “Cerebral Palsy, Athetoid”[TIAB] OR “Athetoid Cerebral Palsy”[TIAB] OR “Cerebral Palsies, Athetoid”[TIAB] OR “Cerebral Palsy, Dyskinetic”[TIAB] OR “Cerebral Palsies, Dyskinetic”[TIAB] OR “Dyskdesfecinetic Cerebral Palsy”[TIAB] OR “Cerebral Palsy, Atonic”[TIAB] OR “Atonic Cerebral Palsy”[TIAB] OR “Cerebral Palsy, Hypotonic”[TIAB] OR “Hypotonic Cerebral Palsies”[TIAB] OR “Hypotonic Cerebral Palsy”[TIAB] OR “Cerebral Palsy, Diplegic, Infantile”[TIAB] OR “Diplegic Infantile Cerebral Palsy”[TIAB] OR “Infantile Cerebral Palsy, Diplegic”[TIAB] OR "Stroke"[Mesh] OR “Stroke”[TIAB] OR “Strokes”[TIAB] OR “Cerebrovascular Accident”[TIAB] OR “Cerebrovascular Accidents”[TIAB] OR “Vascular Accident, Brain”[TIAB] OR “Brain Vascular Accident”[TIAB] OR “Brain Vascular Accidents”[TIAB] OR “Vascular Accidents, Brain”[TIAB] OR “Apoplexy”[TIAB] OR “Cerebrovascular Accident, Acute”[TIAB] OR “Acute Cerebrovascular Accident”[TIAB] OR “Acute Cerebrovascular Accidents”[TIAB] OR “Cerebrovascular Accidents, Acute”[TIAB] OR "Spinal Cord Injuries"[Mesh] OR "Spinal Cord Injuries"[TIAB] OR “Spinal Cord Trauma”[TIAB] OR “Cord Trauma, Spinal”[TIAB] OR “Cord Traumas, Spinal”[TIAB] OR “Spinal Cord Traumas”[TIAB] OR “Trauma, Spinal Cord”[TIAB] OR “Traumas, Spinal Cord”[TIAB] OR “Myelopathy, Traumatic”[TIAB] OR “Myelopathies, Traumatic”[TIAB] OR “Traumatic Myelopathies”[TIAB] OR “Traumatic Myelopathy”[TIAB] OR “Injuries, Spinal Cord”[TIAB] OR “Cord Injuries, Spinal”[TIAB] OR “Cord Injury, Spinal”[TIAB] OR “Injury, Spinal Cord”[TIAB] OR “Spinal Cord Injury”[TIAB] OR “Spinal Cord Transection”[TIAB] OR “Cord Transection, Spinal”[TIAB] OR “Cord Transections, Spinal”[TIAB] OR “Spinal Cord Transections”[TIAB] OR “Transection, Spinal Cord”[TIAB] OR “Transections, Spinal Cord”[TIAB] OR “Spinal Cord Laceration”[TIAB] OR “Cord Laceration, Spinal”[TIAB] OR “Cord Lacerations, Spinal”[TIAB] OR “Laceration, Spinal Cord”[TIAB] OR “Lacerations, Spinal Cord”[TIAB] OR “Spinal Cord Laceration”[TIAB] OR “Post-Traumatic Myelopathy”[TIAB] OR “Myelopathies, Post-Traumatic”[TIAB] OR “Myelopathy, Post Traumatic”[TIAB] OR “Post Traumatic Myelopathy”[TIAB] OR “Post-Traumatic Myelopathies”[TIAB] OR “Spinal Cord Contusion”[TIAB] OR “Contusion, Spinal Cord”[TIAB] OR “Contusions, Spinal Cord”[TIAB] OR “Cord Contusion, Spinal”[TIAB] OR “Cord Contusions, Spinal”[TIAB] OR “Spinal Cord Contusions”[TIAB] OR "Brain Injuries, Traumatic"[Mesh] OR "Brain Injuries, Traumatic"[TIAB] OR “Brain Injury, Traumatic”[TIAB] OR “Traumatic Brain Injuries”[TIAB] OR “Trauma, Brain”[TIAB] OR “Brain Trauma”[TIAB] OR “Brain Traumas”[TIAB] OR “Traumas, Brain”[TIAB] OR “Traumatic Brain Injury”[TIAB] OR “Encephalopathy, Traumatic”[TIAB] OR “Encephalopathies, Traumatic”[TIAB] OR “Traumatic Encephalopathies”[TIAB] OR “Injury, Brain, Traumatic”[TIAB] OR “Traumatic Encephalopathy”[TIAB] OR “Traumatic Brain Injury”[TIAB])

**EMBASE**

('spasticity'/exp OR 'muscle spasticity' OR 'spastic' OR 'spastic disease' OR 'spasticism' OR 'spasticity' OR 'muscle hypertonia'/exp OR 'muscle rigidity'/exp OR 'muscle rigidity' OR 'muscle stiffness' OR 'multiple sclerosis'/exp OR 'cerebral palsy'/exp OR 'cerebrovascular accident'/exp OR 'spinal cord disease'/exp OR 'traumatic brain injury'/exp) AND ('cannabis'/exp OR 'cannabis' OR 'cannabidiol'/exp OR 'cannabidiol' OR 'cannabinoid'/exp OR 'cannabinoid' OR 'cannabinoids' OR 'tetrahydrocannabinol'/exp OR 'tetrahydrocannabinol' OR 'nabiximols'/exp OR 'sativex' OR 'nabilone'/exp OR 'nabilone' OR 'dronabinol'/exp OR 'dronabinol' OR 'cannabigerol'/exp OR 'cannabigerol' OR 'cannabidivarin'/exp OR 'cannabidivarin' OR 'cannabichromene'/exp OR 'cannabichromene' OR 'bedrobinol'/exp OR 'tetrahydrocannabivarin'/exp) AND ('randomized controlled trial'/exp OR 'randomized controlled trial' OR 'cohort analysis'/exp OR 'cohort study' OR 'clinical trial'/exp OR 'clinical trial' OR 'longitudinal study'/exp OR 'longitudinal studies' OR 'longitudinal study')

**LILACS**

(mh:("Espasticidade Muscular") OR mh:("Paralisia Cerebral") OR mh:("Esclerose Múltipla") OR mh:("AVC Embólico") OR ("Muscle Spasticity" OR “spasticity” OR “muscle hypertonia” OR “muscle rigidity” OR "Clasp Knife Spasticity" OR " Clasp-Knife Spasticity" OR "Spastic" OR "Spasticity, Clasp-Knife" OR "Spasticity, Muscle" OR "spastic*" OR "cerebral palsy" OR "Multiple Sclerosis" OR "Stroke" OR "Spinal Cord Injuries" OR "Brain Injuries, Traumatic")) AND (mh:("Cannabis") OR mh:("Canabidiol") OR ("cannabis" OR "cannabidiol" OR "cannabinoids" OR “tetrahydrocannabinol” OR “nabiximols” OR “nabilone” OR “cannabigerol” OR "dronabinol" OR “cannabidivarin” OR “cannabichromene” OR “bedrobinol” OR “tetrahydrocannabivarin”))

**Supplementary Material 2 – Data extraction and imputation procedures for the meta-analysis**

When numerical data were not directly available in the full texts, values were extracted from graphs using the WebPlotDigitizer software in the studies by Collin et al. (2007), Markova et al. (2018), and De Blasiis et al. (2021), as recommended by the *Cochrane Handbook for Systematic Reviews of Interventions* (Sections 6.5.2; 7.7.3).

When only 95% confidence intervals or standard errors were reported, standard deviations were estimated using RevMan, in accordance with the guidance of the *Cochrane Handbook*. (Sections 6.5.2.2 and 6.5.2.3). In situations in which only the standard error was available, the standard deviation was calculated as SD = SE × √n, as applied in the studies by Collin et al. (2010) and Novotna et al. (2011).

When the standard deviation of change was not reported, it was imputed using correlation coefficients derived from the study by Fairhurst et al. (2020), adopting values of 0.45 for the active group (THC:CBD) and 0.30 for the placebo group. This procedure was applied in the studies by Collin et al. (2010), Novotna et al. (2011), Leocani et al. (2015), and Wade et al. (2003), as recommended by the *Cochrane Handbook* (Section 6.5.2.8).

In the absence of follow-up standard deviations, it was assumed that the standard deviation of follow-up measures was similar to that at baseline, when appropriate. This procedure was applied in the study by Leocani et al. (2015), in accordance with guidance from the *Cochrane Handbook* (Section 6.5.2).

**Supplementary Material 3 – Excluded studies in elegibility**

| **Study** | **Citation** | **Reason for exclusion** |
| --- | --- | --- |
| Greenberg, H.S. et al., 1994 | GREENBERG, H. S. et al. Short-term effects of smoking marijuana on balance. Clin Pharmacol Ther, v. 55, n. 3, p. 324–328, 1994. | Wrong study design – comparator group composed of healthy individuals |
| Brady, C. M. et al., 2004 | BRADY, C. M. et al. Cannabis-based extracts for bladder dysfunction. Mult. Scler., v. 10, n. 4, p. 425–433, 2004. | Wrong study design – no comparator group |
| Fox, P. et al., 2004 | FOX, P. et al. Effect of cannabis on tremor in MS. Neurology, v. 62, n. 7, p. 1105–1109, 2004. | Wrong population – presence of spasticity not specified in the methods or results |
| Grotenhermen, F. 2004 | GROTENHERMEN, F. Cannabinoids and muscle spasticity. Evid.-Based Healthc., v. 8, n. 3, p. 159–161, 2004. | Wrong publication type – commentary on another article |
| Notcutt, W. et al., 2004 | NOTCUTT, W. et al. Initial experiences with medicinal cannabis for chronic pain. Anaesthesia, v. 59, n. 5, p. 440–452, 2004. | Wrong population – presence of spasticity not specified in the methods or results |
| Svendsen, K.B. et al., 2004 | SVENDSEN, K. B. et al. Dronabinol reduces central pain in MS? BMJ, v. 329, n. 7460, p. 253, 2004. | Wrong population – presence of spasticity not specified in the methods or results |
| Rog, D. J. et al. 2005 | ROG, D. J. et al. Cannabis-based medicine for central pain. Neurology, v. 65, n. 6, p. 812–819, 2005. | Wrong population – spasticity was an exclusion criterion |
| Svendsen, K.B. et al., 2005 | SVENDSEN, K. B. et al. Synthetic cannabinoid dronabinol on central pain in MS. Ugeskrift for Læger, v. 167, n. 25, p. 2772–2774, 2005. | Wrong population – presence of spasticity not specified in the methods or results |
| Freeman, R.M. et al., 2006 | FREEMAN, R. M. et al. Cannabis on incontinence in MS. Int. Urogynecol. J., v. 17, n. 6, p. 636–641, 2006. | Wrong outcome – outcomes not prespecified in the protocol |
| Maas, A.I. et al., 2006 | MAAS, A. I. et al. Dexanabinol in severe TBI: phase III trial. Lancet Neurology, v. 5, n. 1, p. 38–45, 2006. | Wrong population – presence of spasticity not specified in the methods or results |
| Wade, D.T. et al., 2006 | WADE, D. T. et al. Long-term cannabis use in MS. Mult. Scler., v. 12, n. 5, p. 639–645, 2006. | Wrong study design – single-arm follow-up study (no comparator group) |
| Kavia, R.B.C et al., 2010 | KAVIA, R. B. C. et al. Sativex for bladder in MS. Mult. Scler., v. 16, n. 11, p. 1349–1359, 2010. | Wrong population – presence of spasticity not specified in the methods or results |
| Rintala, D. H. et al., 2010 | RINTALA, D. H. et al. Dronabinol on central neuropathic pain after SCI. Am J Phys Med Rehabil, v. 89, n. 10, p. 840–848, 2010. | Wrong population – unclear from methods and results whether all patients had spasticity |
| Vecchio, D. et al., 2010 | VECCHIO, D. et al. Cannabinoids in multiple sclerosis: A neurophysiological analysis. Acta Neurologica Scandinavica, v. 142, n. 4, p. 333–338, 2020. | Wrong study design – comparator group composed of healthy individuals |
| Weber, M. et al., 2010 | WEBER, M. et al. THC for cramps in ALS. J. Neurol. Neurosurg. Psychiatry, v. 81, n. 10, p. 1135–1140, 2010. | Wrong population – presence of spasticity not specified in the methods or results |
| Katafygiotis, S. et al. 2011 | KATAFYGIOTIS, S. et al. Cannabinoids for urinary symptoms. Neurourol. Urodyn., v. 30, n. 6, p. 846–847, 2011. | Wrong publication type – abstract only |
| Firsching, R. et al., 2012 | FIRSCHING, R. et al. CB1/CB2 agonist in TBI: phase II. J. Neurol. Surg. A, v. 73, n. 4, p. 204–216, 2012. | Wrong population – presence of spasticity not specified in the methods or results |
| Mallik, S. et al., 2013 | MALLIK, S. et al. CUPID MRI sub-study. J. Neurol. Neurosurg. Psychiatry, v. 84, n. 11, 2013. | Wrong publication type – abstract only |
| Rezapour-Firouzi, S. et al., 2013 | REZAPOUR-FIROUZI, S. et al. Lipid enzymes in MS with hemp oil. J. Pure Appl. Microbiol., v. 7, n. 4, p. 2891–2901, 2013. | Wrong population – presence of spasticity not specified in the methods or results |
| Zajicek, J. et al., 2013 | ZAJICEK, J. et al. CUPID trial on dronabinol in MS. Lancet Neurol., v. 12, n. 9, p. 857–865, 2013. | Wrong population – unclear from methods and results whether patients had spasticity |
| Rezapour-Firouzi, S. et al., 2014 | REZAPOUR-FIROUZI, S. et al. Liver enzymes in MS and hemp diet. Complement Ther Med., v. 22, n. 6, p. 986–993, 2014. | Wrong population – presence of spasticity not specified in the methods or results |
| Rezapour-Firouzi, S. et al., 2015 | REZAPOUR-FIROUZI, S. et al. Hemp seed diet in MS. Complement Ther Med., v. 23, n. 5, p. 652–657, 2015. | Wrong population – presence of spasticity not specified in the methods or results |
| Turcotte, D. et al., 2015 | TURCOTTE, D. et al. Nabilone + gabapentin for MS pain. Pain Med., v. 16, n. 1, p. 149–159, 2015. | Wrong population – presence of spasticity not specified in the methods or results |
| Etges, T. et, al. 2016 | ETGES, T. et al. Postmarketing safety registry of Sativex®. Ther. Clin. Risk Manage., v. 12, p. 1667–1675, 2016. | Wrong study design – no comparator group |
| Riva, N et al., 2016 | RIVA, N. et al. CANALS study: cannabis in motor neuron disease. Eur. J. Neurol., v. 23, p. 46, 2016. | Wrong publication type – oral presentation abstract |
| Riva, N et al., 2016 | RIVA, N. et al. CANALS study: cannabis in motor neuron disease. Eur. J. Neurol., v. 23, p. 46, 2016. | Wrong publication type – abstract only |
| Squintani, G. et al., 2016 | SQUINTANI, G. et al. Cortical and spinal excitability in MS and spasticity after cannabinoid spray. J. Neurological Sciences, v. 370, p. 263–268, 2016. | Wrong study design – comparator group composed of healthy individuals |
| Wilsey, B.L et al., 2016 | WILSEY, B. L. et al. Cannabinoid blood concentration and analgesia. J. Pain Res., v. 9, p. 587–598, 2016. | Wrong population – spinal cord injury patients |
| Fairhurst et al., 2020 | FAIRHURST, C. et al. Nabiximols for spasticity in children with cerebral palsy. Dev Med Child Neurol., 2020. | Wrong population – cerebral palsy (not multiple sclerosis) |
| Stefanović et al., 2025 | STEFANOVIĆ, I. et al. Full-spectrum cannabis oil in pediatric spastic cerebral palsy. Neuropediatrics., 2025. | Wrong population – cerebral palsy (not multiple sclerosis) |
| Riva et al., 2018 | RIVA, N. et al. Nabiximols in amyotrophic lateral sclerosis: randomized placebo-controlled trial. Eur J Neurol., 2018. | Wrong population – amyotrophic lateral sclerosis |
| Austin et al., 2022 | AUSTIN, M. et al. Medical cannabis use in ALS: retrospective cohort study. Muscle Nerve., 2022. | Wrong study design – retrospective observational study |
| Marinelli et al., 2022 | MARINELLI, L. et al. Nabiximols for post-stroke spasticity: randomized crossover trial. BMJ Neurology Open., 2022. | Wrong population – post-stroke spasticity |
| Hagenbach et al., 2007 | HAGENBACH, U. et al. Δ9-THC/CBN/CBD extract in spinal cord injury: pilot study. Spinal Cord., 2007. | Wrong population – spinal cord injury |
| Pooyania et al., 2010 | POOYANIA, S. et al. Nabilone for spasticity after spinal cord injury: crossover trial. J Spinal Cord Med., 2010. | Wrong population – traumatic spinal cord injury |
| Petro, 1981 | PETRO, D. et al. Oral THC in multiple sclerosis: pilot trial. Clin Pharmacol Ther., 1981. | Wrong study design – non-randomized pilot study |
| Calabrò et al., 2020 | CALABRÒ, R. et al. Nabiximols plus rehabilitation in MS spasticity: nonrandomized controlled study. J Neurol Sci., 2020. | Wrong study design – non-randomized study |
| Sartori et al., 2021 | SARTORI, A. et al. Nabiximols in routine clinical practice for MS spasticity: retrospective analysis. Mult Scler Relat Disord., 2021. | Wrong study design – retrospective observational study |

**Supplementary Material 4 – Funding sources of the included studies**

Studies were grouped according to the primary declared funder, classified as industry, public, academic, philanthropic, or mixed, as described in the original manuscripts. When applicable, mixed funding or institutional support was explicitly indicated. The classification was based exclusively on the information reported in the included articles.

| **Funding** | **Classification** | **Studies** |
| --- | --- | --- |
| GW Pharmaceuticals plc | Industry | Wade et al., 2003; Wade et al., 2004; Collin et al., 2007; Collin et al., 2010; Notcutt et al., 2011; Novotna et al., 2011; Langford et al., 2012; Vachová et al., 2014 |
| GW Pharmaceuticals + Universidade Sapienza | Mixed (industry + academic) | Aragona et al., 2009; Conte et al., 2009 |
| Society for Clinical Research + Weleda AG | Mixed (industry + association) | Zajicek et al., 2012 |
| Laboratórios Almirall / Almirall | Industry | Leocani et al., 2015; Markova et al., 2018 |
| Echo Pharmaceuticals | Industry | Van Amerongen et al., 2017 |
| Greenwich Biosciences (Jazz Pharmaceuticals) | Industry | Bethoux et al., 2024 |
| Ministério da Saúde, Bem-Estar e Esporte (Países Baixos) | Industry | Killestein et al., 2002 |
| Medical Research Council (MRC) + Multiple Sclerosis Society (UK) | Public / philanthropic | Zajicek et al., 2003 |
| Ministério da Saúde da Suíça | Public | Vaney et al., 2004 |
| NIHR + Medical Research Council (MRC) | Public / philanthropic | Ball et al., 2015 |
| Ministério da Saúde da Dinamarca + fundações | Public / philanthropic | Hansen et al., 2023 |
| Universidade Sapienza de Roma | Academic | Tomassini et al., 2014 |
| University of California – CMCR | Public / Academic | Corey-Bloom et al., 2012 |
| Mashhad University of Medical Sciences | Academic | Mousavi et al., 2025 |
| Not reported | Not reported | Ungerleider et al., 1988; Zajicek et al., 2005; Wissel et al., 2006 |
| No specific funding declared | No specific funding | Blasiis et al., 2021 |

|  |  |  |
| --- | --- | --- |

**Supplementary Material 5 –** Individual results of randomized clinical trials evaluating cannabinoids for the treatment of spasticity in patients with multiple sclerosis, presented according to the assessment instrument used.

| **Study** | **Type of cannabinoid** | **Outcome by assessment instrument** |
| --- | --- | --- |
| Ungerleider 1988 | THC | Non-standardized clinical scale (+) |
| Killestein 2002 | THC; Cannabis extract | Ashworth (-); VAS (-) |
| Wade 2003 | THC; CBD; THC:CBD | NRS (+); Ashworth: **(-)** |
| Zajicek 2003 | THC; Cannabis extract | Ashworth (-); |
| Vaney 2004 | Cannabis extract | Ashworth (-) |
| Wade 2004 | THC:CBD | VAS (+); Ashworth: **(–)** |
| Zajicek 2005 | THC; Cannabis extract | Ashworth (-) |
| Wissel 2006 | Nabilona | Ashworth (-) |
| Collin 2007 | THC:CBD | NRS (-) |
| Aragona 2009 | THC:CBD | VAS (-) |
| Conte 2009 | THC:CBD | VAS (-) |
| Collin 2010 | THC:CBD | NRS(-) |
| Notcutt 2011 | THC:CBD | NRS (-)* |
| Novotná 2011 | THC:CBD | NRS (+); MAS (-) |
| Corey-Bloom 2012 | Smoked cannabis | MAS (+) |
| Langford 2012 | THC:CBD | NRS (-) |
| Zajicek 2012 | THC:CBD | MSSS-88 (+) |
| Tomassini 2014 | THC:CBD | MAS (-) |
| Vachová 2014 | THC:CBD | MAS (–) |
| Ball 2015 | THC (dronabinol) | MSSS-88 (-) |
| Leocani 2015 | THC:CBD | NRS (-); MAS (+); |
| Van Amerongen 2017 | THC (Namisol) | Ashworth (-); NRS (-) |
| Marková 2018 | THC:CBD | NRS (+); MAS (+) |
| Blasiis 2021 | THC:CBD | NRS (+) |
| Hansen 2023 | THC; CBD; THC:CBD | NRS (-); cMAS (-) |
| Bethoux 2024 | THC:CBD | MAS LLMT-6 (-) |
| Mousavi 2025 | CBD | NRS (-) |

(+) statistically significant effect compared with the comparator; (−) no statistically significant effect.

**Abbreviations:** MAS = Modified Ashworth Scale; NRS = Numeric Rating Scale; VAS = Visual Analog Scale; MSSS-88 = Multiple Sclerosis Spasticity Scale–88; cMAS = clinical Modified Ashworth Scale.
 *Randomized withdrawal study; the result reflects maintenance of response rather than an initial efficacy gain.*

**Supplementary Material 6 – Meta-analysis plots and sensitivity analyses (leave-one-out)**

Sensitivity analysis (leave-one-out) of the meta-analysis comparing the THC:CBD extract versus placebo (PBO), with exclusion of the study by Blasiis et al. (2021).

Sensitivity analysis (leave-one-out) of the meta-analysis comparing the THC:CBD extract versus placebo (PBO), with exclusion of the study by Collin et al. (2007).

Sensitivity analysis (leave-one-out) of the meta-analysis comparing the THC:CBD extract versus placebo (PBO), with exclusion of the study by Collin et al. (2010).

Sensitivity analysis (leave-one-out) of the meta-analysis comparing the THC:CBD extract versus placebo (PBO), with exclusion of the study by Leocani et al. (2015).

Sensitivity analysis (leave-one-out) of the meta-analysis comparing the THC:CBD extract versus placebo (PBO), with exclusion of the study by Markova et al. (2018).

Sensitivity analysis (leave-one-out) of the meta-analysis comparing the THC:CBD extract versus placebo (PBO), with exclusion of the study by Novotna et al. (2011).

Sensitivity analysis (leave-one-out) of the meta-analysis comparing the THC:CBD extract versus placebo (PBO), with exclusion of the study by Wade et al. (2003).

Meta-analysis and sensitivity analyses of the effect of the THC:CBD extract compared with placebo on self-reported spasticity in patients with multiple sclerosis after 4 weeks of treatment.

Meta-analysis of the effect of the THC:CBD extract compared with placebo on self-reported spasticity in patients with multiple sclerosis after 4 weeks of treatment.

Sensitivity analysis (leave-one-out) of the meta-analysis comparing the THC:CBD extract versus placebo (PBO), with exclusion of the study by Blasiis et al. (2021).

Sensitivity analysis (leave-one-out) of the meta-analysis comparing the THC:CBD extract versus placebo (PBO), with exclusion of the study by Collin et al. (2007).

Sensitivity analysis (leave-one-out) of the meta-analysis comparing the THC:CBD extract versus placebo (PBO), with exclusion of the study by Leocani et al. (2015).

Sensitivity analysis (leave-one-out) of the meta-analysis comparing the THC:CBD extract versus placebo (PBO), with exclusion of the study by Markova et al. (2018).

Sensitivity analysis (leave-one-out) of the meta-analysis comparing the THC:CBD extract versus placebo (PBO), with exclusion of the study by Novotna et al. (2011).

**Supplementary Material 7 – Network meta-analysis**

Network meta-analysis plots.

Network meta-analysis plots showing the efficacy ranking of interventions for self-reported spasticity, based on SUCRA values. The included interventions were CBD, dronabinol (THC), dronabinol + CBD (fixed combination), CBD-rich extract, THC-rich extract, THC:CBD extract, and placebo.

*Trace plot of the network meta-analysis for self-reported spasticity, demonstrating adequate convergence of the Bayesian model chains.*

Density plot of the network meta-analysis for self-reported spasticity, illustrating the posterior distributions of the effect estimates.

Adverse event ranking of interventions from the network meta-analysis for self-reported spasticity, based on SUCRA values (CBD, dronabinol [THC], dronabinol + CBD [fixed combination], CBD-rich extract, THC-rich extract, THC:CBD extract, and placebo).

*Trace plot of the network meta-analysis for adverse events, demonstrating convergence of the Bayesian model chains.*

Density plot of the network meta-analysis for adverse events, illustrating the posterior distributions of the effect estimates.

Graphical representation of the evidence network of the network meta-analysis of adverse events associated with cannabinoid use for the treatment of spasticity in patients with multiple sclerosis. The nodes represent the evaluated interventions, with size proportional to the number of included participants, and the edges indicate direct comparisons between interventions, with thickness proportional to the number of studies.

League table of the network meta-analysis of adverse events associated with cannabinoid interventions for the treatment of spasticity in patients with multiple sclerosis.

| Ext_cannabis | |  |  |  |  |
| --- | --- | --- | --- | --- | --- |
|  | |  |  |  |  |
| 1.39 (0.35, 6.45) | Ext_CBD |  |  |  |  |
| 0.63 (0.19, 2.17) | 0.45 (0.14, 1.44) | Ext_THC |  |  |  |
| 1.01 (0.41, 2.70) | 0.73 (0.23, 2.15) | 1.59 (0.61, 4.27) | Ext_THC_CBD | |  |
| 1.34 (0.60, 3.25) | 0.99 (0.28, 2.85) | 2.14 (0.84, 5.75) | 1.34 (0.89, 2.05) | Placebo |  |
| 0.98 (0.40, 2.22) | 0.70 (0.17, 2.35) | 1.54 (0.48, 4.37) | 0.96 (0.45, 1.93) | 0.72 (0.37, 1.25) | THC |

**Interventions:** CBD: cannabidiol; Dronabinol: synthetic THC; Dronabinol+CBD: combination of synthetic THC and CBD; Ext_CBD: CBD-rich extract; Ext_THC: THC-rich extract; Ext_THC_CBD: standardized extract containing THC and CBD; PBO: placebo.

**Note:** Odds ratios (OR) with 95% credibility intervals (95% CrI) derived from the network meta-analysis are presented, comparing different cannabinoid formulations and placebo (PBO). Values > 1 indicate a higher risk of adverse events in favor of the intervention listed in the row, whereas values < 1 indicate a higher risk in favor of the intervention listed in the column. Bold values indicate statistically significant comparisons, defined as those whose 95% credibility intervals do not cross the null value (OR = 1).

**Supplementary Material 8 – Risk of bias assessment of the included trials by outcome, according to the Risk of Bias 2.0 (RoB 2.0) tool.**

The outcomes assessed were spasticity (clinical scales), functional capacity and motor function, objective examinations (electromyography, electrical stimulation, and ultrasonography), gait, muscle strength, spasticity-related pain, quality of life, withdrawal due to adverse events, serious adverse events, and adverse events.

| **Change in spasticity (scale)** | | | | | | | |  |
| --- | --- | --- | --- | --- | --- | --- | --- | --- |
| **Study** | **D1** | **DS** | **D2** | **D3** | **D4** | **D5** | **Final** |  |
| Blasiis et al. 2021 |  |  |  |  | 3 | 4 | + |  |
| Leocani, et al. 2015 | 1 |  |  | 6 |  |  | + |  |
| Zajicek et al., 2003 e 2005 |  |  |  |  |  | 5 | ? |  |
| Zajicek et al., 2012 |  |  |  | 6, 7 |  |  | ? |  |
| Wade, et al., 2003 |  |  |  |  |  | 5 | ? |  |
| Vaney, et. al., 2004 |  | 8 |  |  |  | 5 | + |  |
| Ball et al., 2015 |  |  |  | 7 |  |  | ? |  |
| Hansen, et al. 2023 |  |  |  | 6, 7 |  |  | ? |  |
| Corey-Bloom et al., 2012 | 1,2 |  |  |  |  |  | ? |  |
| Wissel, et al., 2006 | 1,2 |  |  |  |  | 5 | ? |  |
| Collin, et al., 2007 | 1 |  |  |  |  | 5 | ? |  |
| Collin, et al., 2010 | 1 |  |  |  |  | 5 | ? |  |
| Langford. et. al, 2012 |  |  |  |  |  |  | - |  |
| Markova, 2018 | 1,2 |  |  |  |  | 5 | ? |  |
| Notcutt, 2011 | 10 |  |  |  |  | 5 | ? |  |
| Novotna, 2011 | 1, 2 |  |  |  |  |  | ? |  |
| Ungerleider, 1988 | 1,2 |  |  |  |  | 5 | ? |  |
| Van Amerongen, et al., 2017 |  |  |  |  |  |  | - |  |
| Wade, et al., 2004 |  |  |  | * |  | 5 | ? |  |
| Tomassini, et al., 2014 | 1,2 |  |  |  |  | 5 | ? |  |
| Vachova, et al., 2014 |  |  |  |  |  | 5 | ? |  |
| Bethoux et al., 2024 |  |  |  |  |  |  | - |  |
| Mousavi et al., 2025 |  |  |  |  |  |  | - |  |
| **Change in functional capacity (scale)/ Change in motor function (scale)** | | | | | | | |  |
| Aragona et al., 2009 | 2 |  |  |  |  | 5 | ? |  |
| Blasiis et al. 2021 |  |  |  |  | 3 | 4 | + |  |
| Zajicek et al., 2012 |  |  |  | 6, 7 |  |  | ? |  |
| Wade, et al., 2003 |  |  |  |  |  | 5 | ? |  |
| Vaney, et. al., 2004 |  | 8 |  |  |  | 5 | + |  |
| Ball et al., 2015 |  |  |  | 7 |  |  | ? |  |
| Hansen, et al. 2023 |  |  |  | 6, 7 |  |  | ? |  |
| Corey-Bloom et al., 2012 | 1 |  |  |  |  | 5 | ? |  |
| Vachova, et al., 2014 |  |  |  |  |  | 5 | ? |  |
| Markova, 2018 | 1,2 |  |  |  |  | 5 | ? |  |
| Wade, et al., 2004 | 10 |  |  | ** |  | 5 | + |  |
| Langford. et. al, 2012 |  |  |  |  |  |  | ? |  |
| Notcutt, 2011 | 10 |  |  |  |  | 5 | ? |  |
| Novotna, 2011 | 1, 2 |  |  |  |  |  | ? |  |
| Collin, et al., 2010 | 1 |  |  |  |  | 5 | ? |  |
| Van Amerongen, et al., 2017 |  |  |  |  |  |  | - |  |
| Mousavi et al., 2025 |  |  |  |  |  |  | - |  |
| Zajicek et al., 2003 |  |  |  |  |  | 5 | ? |  |
| **Change in exams (electromyography, electrical stimulation and ultrasonography)** | | | | | | | |  |
| Conte et al., 2009 | 1 |  |  |  |  | 5 | ? |  |
| Leocani et al., 2015 | 1 |  |  | 6 |  |  | ? |  |
| **Gait** | | | | | | | |  |
| Blasiis et al. 2021 |  |  |  |  |  | 4 | + |  |
| Ungerleider, 1988 | 1,2 |  |  |  |  | 5 | ? |  |
| **Change in muscle strength** | | | | | | | |  |
| Collin, et al., 2007 | 1 |  |  |  |  | 5 | ? |  |
| Notcutt, 2011 | 10 |  |  |  |  | 5 | ? |  |
| Novotna, 2011 | 1, 2 |  |  |  |  |  | ? |  |
| **Change in pain associated with spasticity** | | | | | | | |  |
|  |  |  |  |  |  |  |  |  |
| Conte, et al., 2009 | 1 |  |  |  | 11 | 5 | + |  |
| Wade, et al., 2003 |  |  |  |  | 11 | 5 | ? |  |
| Zajicek et al., 2012 |  |  |  | 6, 7 | 11 |  | ? |  |
| Hansen, et al. 2023 |  |  |  | 6, 7 | 11 |  | ? |  |
| Corey-Bloom et al., 2012 | 1 |  |  |  | 11 | 5 | + |  |
| Collin, et al., 2010 | 1 |  |  |  | 11 | 5 | + |  |
| Wade, et al., 2004 | 10 |  |  | 6 | 11 | 5 | + |  |
| Langford. et. al, 2012 |  |  |  |  | 11 |  | ? |  |
| Markova, 2018 | 1,2 |  |  |  | 11 | 5 | + |  |
| Wissel, et al., 2006 | 1,2 |  |  |  | 11 | 5 | + |  |
| Van Amerongen, et al., 2017 |  |  |  |  | 11 |  | ? |  |
| Mousavi et al., 2025 |  |  |  |  | 11 |  | ? |  |
| **Change in quality of life scores** | | | | | | | |  |
| Aragona et al., 2009 | 2 |  |  |  | 11 | 5 | + |  |
| Hansen, et al. 2023 |  |  |  |  | 11 |  | ? |  |
| Wade, et al., 2003 |  |  |  |  | 11 | 5 | ? |  |
| Ball et. al. 2015 |  |  |  | 7 | 11 |  | ? |  |
| Collin, et al., 2010 | 1 |  |  |  | 11 | 5 | + |  |
| Langford. et. al, 2012 |  |  |  |  | 11 |  | ? |  |
| Markova, 2018 | 1,2 |  |  |  | 11 | 5 | + |  |
| Novotna, 2011 | 1, 2 |  |  |  | 11 |  | + |  |
| Wade, et al., 2004 | 10 |  |  |  | 11 | 5 | + |  |
| **Withdrawals due to adverse effects** | | | | | | | |  |
| Collin, et al., 2007 | 1 |  |  |  |  | 5 | ? |  |
| Collin, et al., 2010 | 1 |  |  |  |  | 5 | ? |  |
| Corey-Bloom et al., 2012 | 1 |  |  |  |  | 5 | ? |  |
| Hansen, et al. 2023 |  |  |  | 6, 7 |  |  | ? |  |
| Langford. et. al, 2012 |  |  |  |  |  |  | - |  |
| Leocani et al., 2015 | 1 |  |  | 6 |  |  | ? |  |
| Markova, 2018 | 1,2 |  |  |  |  | 5 | ? |  |
| Novotna, 2011 | 1, 2 |  |  |  |  |  | ? |  |
| Van Amerongen, et al., 2017 |  |  |  |  |  |  | - |  |
| Vaney, et. al., 2004 |  | 8 |  |  |  | 5 | + |  |
| Wade, et al., 2004 | 10 |  |  |  |  | 5 | ? |  |
| Wade, et al., 2003 |  |  |  |  |  | 5 | ? |  |
| Zajicek et al., 2012 |  |  |  |  |  |  | - |  |
| Zajicek et al., 2003 |  |  |  |  |  | 5 | ? |  |
| Vachova, et al., 2014 |  |  |  |  |  | 5 | ? |  |
| Bethoux et al., 2024 | 1 |  |  |  |  |  | ? |  |
| Mousavi ey al., 2025 |  |  |  |  |  |  | - |  |
| **Characterization of serious adverse effects** | | | | | | | |  |
| Ball et al., 2015 |  |  |  | 7 |  |  | ? |  |
| Collin, et al., 2007 | 1 |  |  |  |  | 5 | ? |  |
| Collin, et al., 2010 | 1 |  |  |  |  | 5 | ? |  |
| Langford et al, 2012 |  |  |  |  |  |  | - |  |
| Notcutt, 2011 | 10 |  |  |  |  | 5 | ? |  |
| Zajicek et al., 2003 e 2005 |  |  |  |  |  | 5 | ? |  |
| Bethoux et al., 2024 | 1 |  |  |  |  |  | ? |  |
| Markova et al., 2018 | 1,2 |  |  |  |  | 5 | ? |  |
| Vachova, et al., 2014 |  |  |  |  |  | 5 | ? |  |
| Vaney et al., 2004 |  | 8 |  |  |  | 5 |  |  |
| Hansen et al., 2003 |  |  |  | 6, 7 |  |  | ? |  |
| Corey-bloom et al., 2012 | 1 |  |  |  |  | 5 | ? |  |
| Van amerongen et al., 2017 |  |  |  |  |  |  | - |  |
| Zajicek et al., 2012 |  |  |  |  |  |  | - |  |
| Riva et al., 2018 |  |  |  |  |  |  | - |  |
| Leocani et al., 2015 | 1 |  |  | 6 |  |  | ? |  |
| Novotna et al., 2011 | 1, 2 |  |  |  |  |  | ? |  |
| Aragona et al., 2009 | 2 |  |  |  |  | 5 | ? |  |
| Killestein et al., 2002 | 1 |  |  |  |  | 5 | ? |  |
| **Characterization of adverse effects** | | | | | | | |  |
| Aragona et al., 2009 | 2 |  |  |  |  | 5 | ? |  |
| Ball et al., 2015 |  |  |  | 7 |  |  | ? |  |
| Blasiis, 2021 |  |  |  |  |  |  | ? |  |
| Collin, et al., 2007 | 1 |  |  |  |  | 5 | ? |  |
| Collin, et al., 2010 | 1 |  |  |  |  | 5 | ? |  |
| Conte, et al., 2009 | 1 |  |  |  |  | 5 | ? |  |
| Corey-Bloom et al., 2012 | 1 |  |  |  |  | 5 | ? |  |
| Leocani, et al. 2015 | 1 |  |  | 6 |  |  | ? |  |
| Notcutt, 2011 | 10 |  |  |  |  | 5 | ? |  |
| Novotna, 2011 | 1, 2 |  |  |  |  |  | ? |  |
| Ungerleider, 1988 | 1,2 |  |  |  |  | 5 | ? |  |
| Van Amerongen et al., 2017 |  |  |  |  |  |  | - |  |
| Vaney, et. al., 2004 |  | 8 |  |  |  | 5 | + |  |
| Wade, et al., 2003 |  |  |  |  |  | 5 | ? |  |
| Wade, et al., 2004 | 10 |  |  |  |  | 5 | ? |  |
| Wissel, et al., 2006 | 1,2 |  |  |  |  | 5 | ? |  |
| Zajicek et al., 2003 |  |  |  |  |  | 5 | ? |  |
| Zajicek et al., 2005 |  |  |  |  |  | 5 | ? |  |
| Zajicek et al., 2012 |  |  |  |  |  |  | - |  |
| Tomassini, et al., 2014 | 1,2 |  |  |  |  | 5 | ? |  |
| Vachova, et al., 2014 |  |  |  |  |  | 5 | ? |  |
| Bethoux et al., 2024 | 1 |  |  |  |  |  | ? |  |

**Legend:** 1 Randomization mentioned but without adequate description of the method; 2 demographic data not reported; 3 subjective outcome assessed by a non-blinded participant; 4 use of multiple outcome measures with unclear results; 5 study protocol not available; 6 exclusion of more than 20% of participants from the final analysis; 7 disproportionate loss of outcomes between groups; 8 inadequate washout period (3 days); 9 absence of direct comparison between intervention and placebo due to baseline differences, with comparison between placebo in the blinded phase and intervention in the open-label phase; 10 relevant baseline differences between groups; 11 outcomes based on subjective self-report, with potential influence of participant expectations or prior experience. *Significant data loss for VAS; not observed for the Ashworth scale. **Significant data loss for the 10MWT (10-meter walk test) and NHPT (Nine-Hole Peg Test); not observed for the Barthel Index (ADL). **Overall risk of bias classification:** low risk of bias (–); some concerns (?); high risk of bias (+).

**Supplementary Material 9. Certainty of evidence**

Assessment of the certainty of evidence for the effect of THC:CBD compared with placebo on self-reported spasticity (NRS) in patients with multiple sclerosis, according to the GRADE system.

| **GRADE domain** | **Judgment** | **Justification** |
| --- | --- | --- |
| Risk of bias | ↓ Downgraded 1 level | Predominance of studies at moderate or high risk for subjective outcomes. One trial has important methodological limitations (crossover design, risk of carryover). |
| Inconsistency | ↓ Downgraded 1 level | Relevant statistical heterogeneity (I² = 53.3%) and lack of overlap of confidence intervals across studies, indicating true variation among results. |
| Indirectness | Not downgraded | Population, intervention, and comparator are directly applicable to the research question. |
| Imprecision | Not downgraded | The confidence interval around the pooled effect is relatively precise and the total sample size is adequate. |
| Publication bias | Not assessed | Not assessed due to the limited number of included studies. |
| Overall certainty of evidence | LOW | Downgraded due to risk of bias and inconsistency. |

Certainty of evidence assessment of the network meta-analysis comparisons for self-reported spasticity in multiple sclerosis, according to the CINeMA framework. Certainty of evidence was rated considering within-study bias, reporting bias, indirectness, imprecision, heterogeneity, and incoherence

| **Comparison** | **Number of studies** | **Within-study bias** | **Reporting bias** | **Indirectness** | **Imprecision** | **Heterogeneity** | **Incoherence** | **Confidence rating** |
| --- | --- | --- | --- | --- | --- | --- | --- | --- |
| CBD:Dronabinol | 1 | Some concerns | Low risk | Some concerns | Major concerns | No concerns | No concerns | Low |
| CBD:Dronabinol + CBD | 1 | Some concerns | Low risk | Some concerns | Major concerns | No concerns | No concerns | Low |
| CBD:PBO | 1 | Some concerns | Low risk | Some concerns | Major concerns | No concerns | No concerns | Low |
| Dronabinol:Dronabinol + CBD | 1 | Some concerns | Low risk | Some concerns | Major concerns | No concerns | No concerns | Low |
| Dronabinol:PBO | 1 | Some concerns | Low risk | Some concerns | Major concerns | No concerns | No concerns | Low |
| Dronabinol + CBD:PBO | 1 | Some concerns | Low risk | Some concerns | Major concerns | No concerns | No concerns | Low |
| Ext CBD:Ext THC | 1 | Some concerns | Low risk | Some concerns | Major concerns | No concerns | No concerns | Low |
| Ext CBD:Ext THC and CBD | 1 | Some concerns | Low risk | Some concerns | Major concerns | No concerns | No concerns | Low |
| Ext CBD:PBO | 1 | Some concerns | Low risk | Some concerns | Major concerns | No concerns | No concerns | Low |
| Ext THC:Ext THC and CBD | 1 | Some concerns | Low risk | Some concerns | Major concerns | No concerns | No concerns | Low |
| Ext THC:PBO | 1 | Some concerns | Low risk | Some concerns | Major concerns | No concerns | No concerns | Low |
| Ext THC and CBD:PBO | 7 | Some concerns | Low risk | No concerns | No concerns | Major concerns | No concerns | Low |
| CBD:Ext CBD | 0 | Some concerns | Low risk | Some concerns | Major concerns | No concerns | No concerns | Low |
| CBD:Ext THC | 0 | Some concerns | Low risk | Some concerns | Major concerns | No concerns | No concerns | Low |
| CBD:Ext THC and CBD | 0 | Some concerns | Low risk | Some concerns | Major concerns | No concerns | No concerns | Low |
| Dronabinol:Ext CBD | 0 | Some concerns | Low risk | Some concerns | Major concerns | No concerns | No concerns | Low |
| Dronabinol:Ext THC | 0 | Some concerns | Low risk | Some concerns | Major concerns | No concerns | No concerns | Low |
| Dronabinol:Ext THC and CBD | 0 | Some concerns | Low risk | Some concerns | Major concerns | No concerns | No concerns | Low |
| Dronabinol + CBD:Ext CBD | 0 | Some concerns | Low risk | Some concerns | Major concerns | No concerns | No concerns | Low |
| Dronabinol + CBD:Ext THC | 0 | Some concerns | Low risk | Some concerns | Major concerns | No concerns | No concerns | Low |
| Dronabinol + CBD:Ext THC and CBD | 0 | Some concerns | Low risk | Some concerns | Major concerns | No concerns | No concerns | Low |

Assessment of the certainty of evidence for the network meta-analysis comparisons of adverse events associated with cannabinoid interventions in the treatment of spasticity in patients with multiple sclerosis, according to the CINeMA framework. The certainty of the evidence was classified considering within-study risk of bias, reporting bias, indirectness, imprecision, heterogeneity, and incoherence.

| **Comparison** | **Number of studies** | **Within-study bias** | **Reporting bias** | **Indirectness** | **Imprecision** | **Heterogeneity** | **Incoherence** | **Confidence rating** |
| --- | --- | --- | --- | --- | --- | --- | --- | --- |
| Ext CBD:Ext THC | 1 | Some concerns | Low risk | Some concerns | Major concerns | No concerns | No concerns | Low |
| Ext CBD:Ext THC and CBD | 1 | Some concerns | Low risk | Some concerns | Major concerns | No concerns | No concerns | Low |
| Ext CBD:Placebo | 1 | Some concerns | Low risk | Some concerns | Major concerns | No concerns | No concerns | Low |
| Ext THC:Ext THC and CBD | 1 | Some concerns | Low risk | Some concerns | Major concerns | No concerns | No concerns | Low |
| Ext THC:Placebo | 1 | Some concerns | Low risk | Some concerns | Major concerns | No concerns | No concerns | Low |
| Ext THC and CBD:Placebo | 8 | Some concerns | Low risk | No concerns | No concerns | Major concerns | No concerns | Low |
| Ext cannabis:Placebo | 1 | Some concerns | Low risk | No concerns | Major concerns | No concerns | No concerns | Low |
| Ext cannabis:THC | 1 | Some concerns | Low risk | No concerns | Major concerns | No concerns | No concerns | Low |
| Placebo:THC | 3 | Some concerns | Low risk | No concerns | Major concerns | No concerns | No concerns | Low |
| Ext cannabis:Ext CBD | 0 | Some concerns | Low risk | No concerns | Major concerns | No concerns | No concerns | Low |
| Ext CBD:THC | 0 | Some concerns | Low risk | No concerns | Major concerns | No concerns | No concerns | Low |
| Ext cannabis:Ext THC | 0 | Some concerns | Low risk | No concerns | Major concerns | No concerns | No concerns | Low |
| Ext THC:THC | 0 | Some concerns | Low risk | No concerns | Major concerns | No concerns | No concerns | Low |
| Ext cannabis:Ext THC and CBD | 0 | Some concerns | Low risk | No concerns | Major concerns | No concerns | No concerns | Low |
| Ext THC and CBD:THC | 0 | Some concerns | Low risk | No concerns | Major concerns | No concerns | No concerns | Low |
